# Patterns and drivers of evapotranspiration in South American wetlands

**DOI:** 10.1038/s41467-023-42467-0

**Published:** 2023-10-20

**Authors:** Ayan Santos Fleischmann, Leonardo Laipelt, Fabrice Papa, Rodrigo Cauduro Dias de Paiva, Bruno Comini de Andrade, Walter Collischonn, Marcelo Sacardi Biudes, Rafael Kayser, Catherine Prigent, Eric Cosio, Nadja Gomes Machado, Anderson Ruhoff

**Affiliations:** 1 Instituto de Desenvolvimento Sustentável Mamirauá, Tefé, Amazonas Brazil; 2 Instituto de Pesquisas Hidráulicas (IPH), Universidade Federal do Rio Grande do Sul (UFRGS), Porto Alegre, Brazil; 3 Université de Toulouse, LEGOS (IRD, CNRS, CNES, UPS), Toulouse, France; 4 Universidade de Brasília (UnB), IRD, Instituto de Geociências, Brasília, Brazil; 5 Instituto de Física, Universidade Federal de Mato Grosso (UFMT), Cuiabá, Brazil; 6 LERMA, CNRS, Observatoire de Paris, Paris, France; 7 Instituto para la Naturaleza, Tierra y Energía (INTE), Pontificia Universidad Católica del Perú, Lima, Perú; 8 Instituto Federal de Mato Grosso (IFMT), Cuiabá, Brazil

**Keywords:** Hydrology, Environmental impact, Hydrology

## Abstract

Evapotranspiration (ET) is a key process linking surface and atmospheric energy budgets, yet its drivers and patterns across wetlandscapes are poorly understood worldwide. Here we assess the ET dynamics in 12 wetland complexes across South America, revealing major differences under temperate, tropical, and equatorial climates. While net radiation is a dominant driver of ET seasonality in most environments, flooding also contributes strongly to ET in tropical and equatorial wetlands, especially in meeting the evaporative demand. Moreover, significant water losses through wetlands and ET differences between wetlands and uplands occur in temperate, more water-limited environments and in highly flooded areas such as the Pantanal, where slow river flood propagation drives the ET dynamics. Finally, floodplain forests produce the greatest ET in all environments except the Amazon River floodplains, where upland forests sustain high rates year round. Our findings highlight the unique hydrological functioning and ecosystem services provided by wetlands on a continental scale.

## Abstract (web summary)

Wetlands can affect regional climate by altering surface-atmosphere interactions. This paper investigates drivers and patterns of evapotranspiration in South American wetlands, from the Amazon floodplains to the large Pantanal system.

## Introduction

**Fig. 1:**
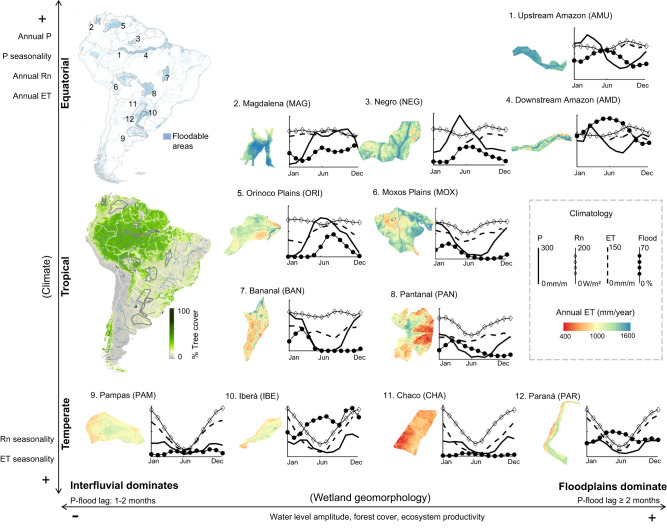
Evapotranspiration patterns across South American wetland complexes. Long-term average evapotranspiration (ET) maps are presented together with climatology of precipitation (P), ET, net radiation (Rn) and flood fraction (Flood). The location of the wetlands in South America are presented together with the continent tree cover map. The wetlands are organized following gradients of climate (from temperate to equatorial) and wetland geomorphology (from interfluvial wetlands to river floodplains, which are more coupled to adjacent rivers). All *Y*-axes have the same scale, with values provided in the dashed box in the right. Tree cover from MOD44B Version 6 Vegetation Continuous Fields product for 2010, available at https://lpdaac.usgs.gov/products/mod44bv006/. Because of persistent cloud cover in the Amazon, ET was not estimated for the months of January to May; however, the available period is representative of the flood maximum and minimum stages, enabling us to understand the seasonal dynamics of ET in this region, while the small ET amplitude in Amazon enables us to estimate its annual rate.

Wetlands support diverse and complex ecosystems worldwide, offering important environmental and societal benefits. They play a critical role in providing freshwater and food, regulating climate, mitigating floods, sequestering carbon, and supporting biodiversity^1,2^. Approximately 12% of South America is covered by wetlands^3^, including the massive wetland systems of the Amazon and the Pantanal^4–6^ (Fig. 1), which encompass a great variety of climates from equatorial to tropical and temperate. Nearly 20% of the South American wetlands are protected today^7^, and recent anthropogenic pressures, such as deforestation, fires, waterway development, climate change, and dam building, have highlighted the need for a better comprehension of ecosystem services and sustainable management of these areas^6,8–10^.

While South America is known as the “fluvial continent” for its large rivers and floodplains, interfluvial wetland complexes are also found across it. These interfluvial complexes are often associated with particular geomorphic settings and savanna or grassland vegetation^2, 6^, although they may also occur in forested swamps^11^. Interfluvial areas include tropical, hyperseasonal ecosystems, where non-forest vegetation has adapted to cope with a soil that ranges from completely dry to fully saturated^5,12,13^, which poses many survival challenges for plants given typical shallow roots in waterlogged soils^14,15^. On the other hand, in the highly dynamic river floodplains, the floristic composition is driven by sedimentation processes and the channel-upland flooding gradient^16^; thus, grasses dominate the most floodable areas, while trees flourish in the less floodable areas—in the cases of the Paraná and Amazon rivers, they remain under water less than 210–270 days per year^17,18^.

Evapotranspiration (ET), a key flux linking surface and atmospheric energy budgets, is also the main consumer of incoming energy and water in wetlands. At the regional landscape level (i.e., the wetlandscape^19^), the existence of large wet surfaces has the potential to affect the partition of available energy into sensible and latent heat, influencing not only local temperature and water quality but also the regional atmospheric boundary layer^20^, the vertical transport of heat and water vapor in the atmosphere, and local-to-regional atmospheric circulation^21–23^. The river or wetland breeze effect, observed, for instance, in the Central Amazon^24,25^, has been suggested to suppress precipitation over flooded areas and initiate convection over wetland edges^22,26^. However, the role of wetland systems on regional to continental atmospheric circulation remains poorly understood, and efforts to develop a global theory of wetland ET dynamics involving the co-evolution between climate, soil, flooding mechanisms, and vegetation is imperative^27^. A proper consideration of wetland hydrological processes within land surface models simulating regional to global climate similarly requires improved physical representations and parameterizations^28^, especially to assess future—and, as yet, uncertain—climate change impacts on wetland hydrology^29^. Such efforts are necessary to complement regional studies related to hydrological changes in South American wetlands^30,31^ and to advance toward a continental-scale quantification of wetland ecosystem services.

To date, few studies have been performed on the ET of South American wetlands^32^, and the few studies that do exist have focused on individual wetlands and local scales, especially parts of the Amazonian and Pantanal wetlands^21,33–35^, which hampers comparisons. These wetland complexes encompass a mixture of both floodplains and interfluvial systems, and from a large-scale perspective only a few areas can be considered as purely floodplain or interfluvial. A comparative hydrology approach involving multiple wetlands, as well as the wetland and its adjacent uplands^36^, arises as a promising framework to understand ET in wetlandscapes across multiple climates and biomes. In doing so, the framework has the potential to facilitate a consistent understanding of the role of various environmental drivers (e.g., precipitation, flooding, available energy, and vapor pressure deficit) and to enable predictions regarding these areas’ responses to ongoing environmental changes^37^. Such comparative hydrology can be undertaken based on remote sensing techniques, which are powerful tools for wetland ET monitoring. This is especially true for diagnostic models that are based on the land surface temperature (LST), which can be coupled with cloud computation frameworks, providing long-term and consistent ET spatial patterns^38,39^. While cloud computation has engendered groundbreaking advances in wetland hydrology by enhancing the understanding of flooding processes in large areas^40^, here we go further by analyzing the spatio-temporal dynamics of ET in 12 large wetland complexes in South America (Fig. 1). These areas were selected as representative of the major wetland systems in the continent, encompassing a broad range of climates, biomes and geomorphological settings. We employ a Google Earth Engine algorithm based on the Surface Energy Balance Algorithm for Land (SEBAL)^41^ model and Moderate Resolution Imaging Spectroradiometer (MODIS) imagery to generate monthly ET estimates, which are jointly analyzed with a state-of-the-art inundation dataset^42^ for the period of 2000–2015. Our objectives are to understand the long-term patterns of wetland ET across the continent, its seasonal patterns and environmental drivers. Regarding floodplain environments, we investigate the role of flood propagation on floodplain ET and how ET rates change across different South American floodplain forests. Thus, this study helps unraveling the patterns and drivers of wetland ET, the effects of wetlands on regional climate, and the general understanding of the ecosystem services they provide across various biomes and geomorphic settings along the continent.

## Results

### Long-term patterns of wetland evapotranspiration across climates

**Fig. 2:**
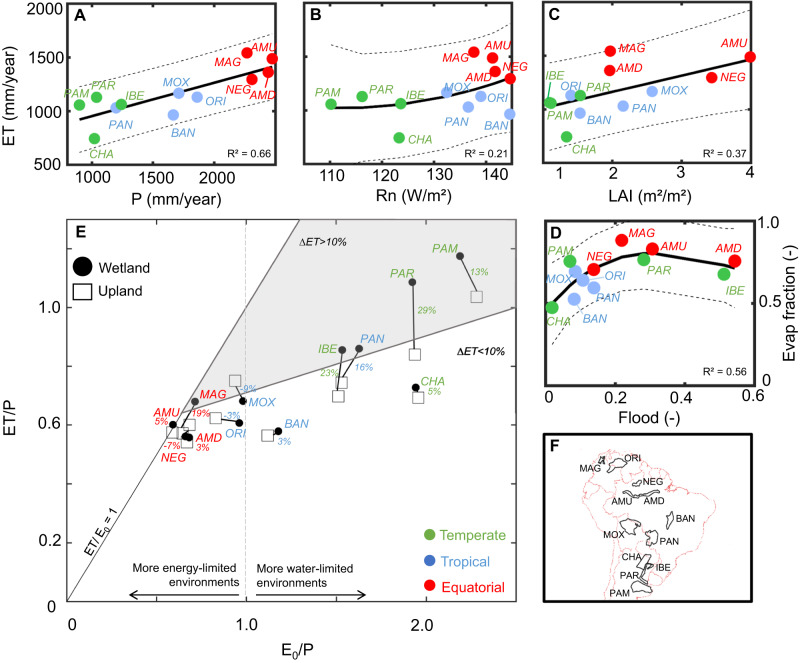
Relationships between long-term mean evapotranspiration and environmental drivers in South American wetlands. Relationships between long-term mean evapotranspiration and annual average (**A**) precipitation (P), (**B**) net radiation (Rn) and (**C**) leaf area index (LAI), and (**D**) between evaporative and flood fractions. Polynomial fits are presented together with the 95% confidence interval. **E** Budyko-like framework relating the long-term evaporative index (ET/P) with the aridity index (E_0_/P), where E_0_/P > 1 refers to more water-limited environments, and E_0_/P < 1 to more energy-limited ones. Values are presented for wetlands (black circles) and the adjacent uplands (black squares) (see the “Methods” section for definition of the areas). The wetland-upland long-term differences are shown as numbers between wetland and upland symbols for each wetland, and the dark gray area refers to areas with differences higher than 10%. Each wetland area refers to its foodable areas and not to its whole catchment, as in the original Budyko framework, so that evaporative index values higher than unity may indicate incoming water from out of the analyzed area. **F** Location of the 12 wetland complexes.

The long-term patterns of ET in South American wetlands follow a climate gradient (Fig. 1). The combination of high precipitation and available energy (assessed here as net radiation; Rn) in equatorial wetlands (Fig. 2A, B) produces the highest annual ET rates (1296–1542 mm/year), and these areas also exhibit the greatest leaf area index values (Fig. 2C). The lowest annual ET rates occur in the temperate wetlands (743–1128 mm/year). While annual Rn is relatively similar between equatorial and tropical wetlands, the higher water availability (precipitation) leads to higher ET in the former. In turn, the fraction of available energy that is converted into ET depends on surface water availability, i.e., the extent to which incoming waters accumulate on the terrain surface. Our regional-scale analysis indicates that, the more flooding the wetland presents, the higher is the evaporative fraction, independent of climate type (Fig. 2D). Examining regional flood fraction values that exceed 0.3, however, reveals that the evaporative fraction reaches a plateau around 0.7–0.8. The fraction of precipitation that becomes evapotranspiration (ET/P) ranges from 0.5–0.7 in equatorial and most tropical wetlands to greater than 0.8 in temperate ones, with the highest values in the Pampas and Paraná floodplains (Fig. 2E). In the assessed region of the Pampas, relatively little runoff is routed out of the wetland through a consolidated river drainage network, and almost all precipitation turns into ET. An ET/P ratio greater than unity for the Pampas suggests a long-lasting effect within the system that is associated with groundwater storage^43^. Meanwhile, the assessed Paraná floodplain receives water from the upstream basin, and total water inflow exceeds precipitation.

Comparing ET in wetlands and adjacent uplands at the regional scale, we uncover greater differences among temperate wetlands, which are located within more water-limited environments (E_0_/P > 1, where E_0_ stands for atmospheric evaporative demand, indicated here by the reference ET), with values reaching 29%, 23%, 13%, and 5% for the Paraná, Iberá, Pampas, and Chaco wetlands, respectively (Fig. 2E). For instance, for the case of Paraná river, this means that the floodplain inundation due to waters coming from upstream may increase the annual average ET by around 250 mm/year in relation to uplands (see monthly values for other wetlands in Supplementary Fig. S1), i.e., a latent heat flux difference of around 20 W/m². The increased surface water availability in these areas enables them to meet the evaporative demand, i.e., higher ET/E_0_ values in wetlands than uplands. In contrast, the ET/E_0_ rate is close to unity (i.e., points close to the 1:1 line in Fig. 2E) for both uplands and wetlands in the equatorial regions. Flooding in large portions of the equatorial Magdalena Mompós depression and the tropical Pantanal wetlands (Fig. S2) produces significant wetland-upland differences (19% and 16%, respectively). Flooding in these areas stems from a combination of geomorphological processes (i.e., depressions associated with sedimentary basins) and large river inflows (the Magdalena and Paraguay rivers, respectively) within a mostly non-forest vegetation landscape. In contrast, the equatorial wetland forest in the Amazon floodplain (both upstream and downstream areas, as depicted in Fig. 1) exhibits only small differences (3–5%) because the large upland forest (“*terra-firme*”) maintains high ET rates throughout the year. The small wetland-upland ET difference (3–5%) is due to open water evaporation, and this effect may be further enhanced by reduced precipitation in the Amazonian floodplains compared to the uplands (−5%^24^), which increases energy availability in the floodplains because of decreased cloud cover. On the other hand, some tropical hyperseasonal systems (Orinoco, Negro interfluvial areas, Moxos, and parts of Bananal), associated with different degrees of mixed herbaceous and woody vegetation, are partly surrounded by upland forested areas. In this case, while the flooded savannas exhibit higher ET rates than do the adjacent non-flooded savannas, the surrounding forested areas appear to maintain high ET rates year-round and therefore exhibit annual ETs that exceed those of the floodable savannas (Fig. S3).

### Seasonal patterns of wetland evapotranspiration and its environmental drivers

**Fig. 3:**
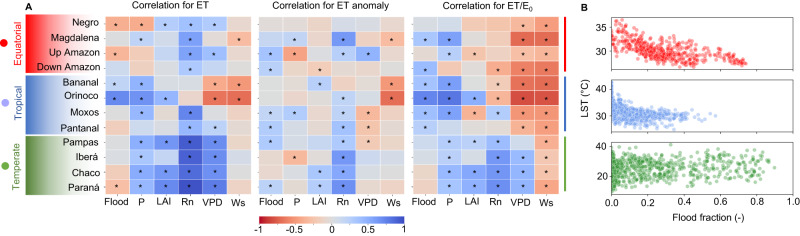
Relationships between monthly evapotranspiration and environmental drivers in South American wetlands. **A** The assessed evaporation-related variables are evapotranspiration (ET), ET anomalies (i.e., seasonal effects are removed), and actual evapotranspiration/evaporative demand ratio (ET/E_0_). The assessed environmental variables are land surface temperature (LST), flood fraction (Flooding), precipitation (P), leaf area index (LAI), net radiation (Rn), vapor pressure deficit (VPD) and wind speed (Ws). The table colors refer to Pearson correlation values. **B** Relationship between monthly LST and flooding. The colors refer to climate type (temperate, tropical and equatorial). *Significant correlations with *P* < 0.01.

We investigate the role of environmental drivers on ET dynamics by correlating monthly ET estimates with six main drivers: flooding, precipitation, leaf area index, Rn, vapor pressure deficit, and wind speed (Fig. 3A; see detailed correlation matrices and scatterplots in Figs. S4 and S5). While precipitation is identified as the main driver of annual ET magnitude across the wetlands (Fig. 2), the available energy (Rn) is the main driver of ET seasonality for equatorial (high water availability throughout the year) and temperate wetlands (high Rn seasonality). In temperate climates, the wet season is in phase with Rn, producing the highest ET rates in the austral summer (Fig. 1). In turn, the tropical wetlands face a dry season water deficit; thus, water availability (measured via both flooding and precipitation variables) complements Rn as a major ET driver.

Wetland LST exhibits strong variation across the continent (Fig. 3B). While it is mainly driven by flooding in equatorial wetlands (a strong correlation between flooding and LST), a different pattern occurs in the temperate wetlands, where the strong Rn seasonality is responsible for the large LST amplitude (Fig. S4). The tropical wetlands exhibit an intermediate pattern, with both Rn and flooding moderately correlated with LST. These differences have major implications for ET dynamics.

**Fig. 4:**
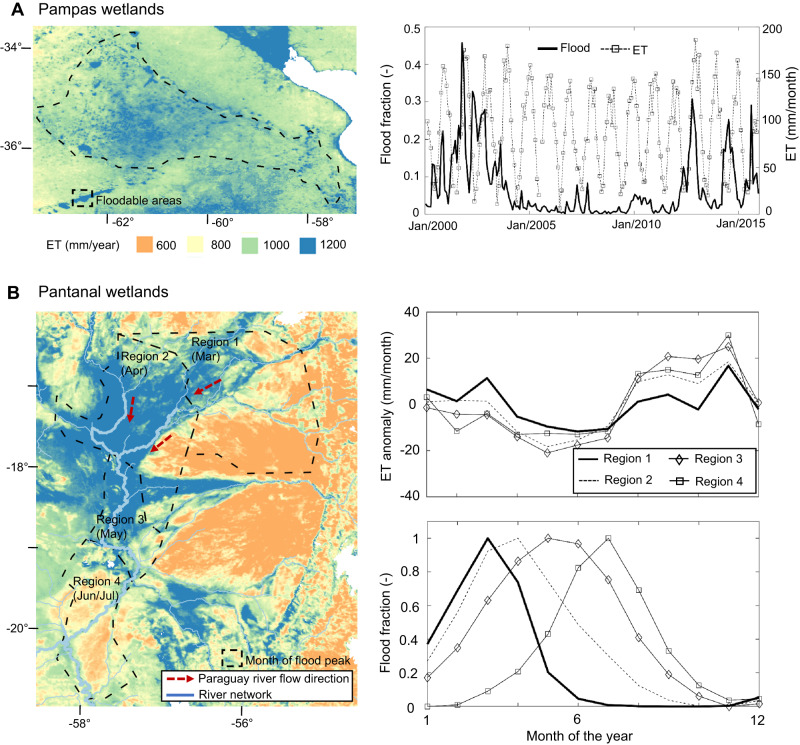
The role of flooding on evapotranspiration spatial pattern. **A** The interannual variation of floods in the Pampas wetlands in Argentina relates to evapotranspiration (ET) patterns. The left figure presents the annual evapotranspiration map, with the floodable areas highlighted, and the right figure shows 15 years of flood fraction and absolute monthly evapotranspiration. **B** Flood propagation affects ET at the seasonal time scale in the Pantanal wetlands. The annual evapotranspiration map is presented in the left figure, together with the location of the four regions of equal month of flood peak (from March to June/July), while the figures in the right column show the climatology of monthly ET (anomaly values) and flood fraction for the four regions.

While, in average years, ET seasonality follows strong Rn variation, years with anomalous flooding lead to anomalously high ET in many South American wetlands (see the correlation for ET anomaly in Fig. 3). This is the case of the Argentinian Pampas, which is characterized by an erratic interannual flooding pattern, with alternately flood-rich (2000–2004 and 2011–2015) and flood-poor periods (2005–2010). While the highest ET rates occurred in the flood years (Fig. 4A), high ET values persisted some years after the main flooding period (2000–2004), indicating long-lasting effects on groundwater storage in the Pampas^43^.

A strong correlation between flooding and ET/E_0_ for many wetlands (Fig. 3) also corroborates the finding of our long-term scale analysis, i.e., that the flooding process generally enables the water supply to meet the evaporative demand. In the downstream areas of the Amazon and the Magdalena and the hyperseasonal wetlands (Fig. S6), the highest ET/E_0_ values occur during the flood peak. In the temperate wetlands, however, ET/E_0_ is mainly driven by Rn. Temperate wetlands exhibit the greatest annual amplitude and the highest monthly rates (e.g., 170 mm/month in December in Iberá; Fig. 1), confirming the hypothesis that grassland wetlands have ET rates as high as forested ones in some months^44^. In turn, the equatorial wetlands exhibit nearly constant ET rates (from 100 to 130 mm/month in the two Amazon floodplain areas assessed; see Fig. 1). Heavy cloud cover in the equatorial Amazon wetlands prevents available energy rates from increasing, especially during the wet season. Months with more flooding are associated with the smallest wetland-upland differences between hyperseasonal wetlands (Negro interfluvial, Orinoco, Moxos, and Bananal) and the upland forested areas that partly cover their surroundings. However, the differences increase during the dry season, with the forests exhibiting higher ET (Figs. S1 and S7). During the wet season, the differences between the flooded savannas and non-flooded forests likewise decrease. Conversely, in the Amazon River floodplains, greater flooding produces a greater difference since uplands are relatively dry when water levels in floodplains are at maximum.

### The role of flood propagation on floodplain evapotranspiration

In terms of flooding mechanisms, inland wetlands can be classified into (1) interfluvial wetlands, which are associated with local runoff and vertical hydrological processes (endogenous processes), (2) river floodplains, where flooding is related to the overbank transfer of waters from upstream areas (exogenous processes), and (3) a combination of both^6, 45^. The flood wave propagation along river floodplains produces a delay of many months between maximum precipitation and flooding at the farthest downstream reaches of the Paraná (2 months), the Amazonian (3 months), and the Pantanal (6 months) wetlands. These delays derive from a combination of vertical soil wetting, which can produce a delay of up to two months, as seen in the more interfluvial wetlands in Fig. 1, and flood wave translation, which can lead to longer delays depending on river hydrodynamics. We demonstrate that flood propagation largely affects the ET dynamics in the Pantanal, which experiences more than 100,000 km² of flooding annually^5^. While precipitation peaks in January across the entire region, the month of maximum flooding varies from March in the upstream reaches to July in the downstream ones (Fig. 4B). ET climatological behavior is driven by the complementary role of flooding and evaporative demand, which reaches its maximum between August and November in the entire region, according to vapor pressure deficit and wind speed patterns (Fig. S8). Consequently, the upstream regions that experience their flood peak in March (i.e., Region 1 in Fig. 4B) exhibit ET peaks in both March and November. Conversely, the downstream regions, where evaporative demand and surface water availability due to floodplain inundation are in phase, reach their annual ET peak between August and November. The higher ET rates in the downstream regions are likely associated with open water evaporation. In turn, the Pantanal’s unique geomorphology causes longer delays in its flood wave translation compared to the other two large floodplains addressed here (the Amazon and the Paraná). However, the effects of flood propagation on ET dynamics are also evident in these wetlands, where anomalous ET rates are strongly correlated with periods of anomalous flooding (Fig. 3).

### Evapotranspiration of floodplain forests across biomes

The partition of wetland ET into vegetation transpiration and open water and soil evaporation is difficult to disentangle. Open water evaporation tends to increase with surface water availability and can potentially offset plant transpiration, which may, in turn, be reduced by flooding due to anoxic or hypoxic conditions, an increase of toxic compounds, or a decrease in the availability of nutrients^46–48^. These effects can induce stomatal closure, while flood adaptation measures, such as adventitious roots and aerenchyma, can, in contrast, increase stomatal conductance during flood peaks^49,50^. The total canopy conductance depends on stomata opening and total leaf area; therefore, the various adaptation strategies plants use to cope with alternating cycles of flooding and drying ultimately determine transpiration seasonality.

**Fig. 5:**
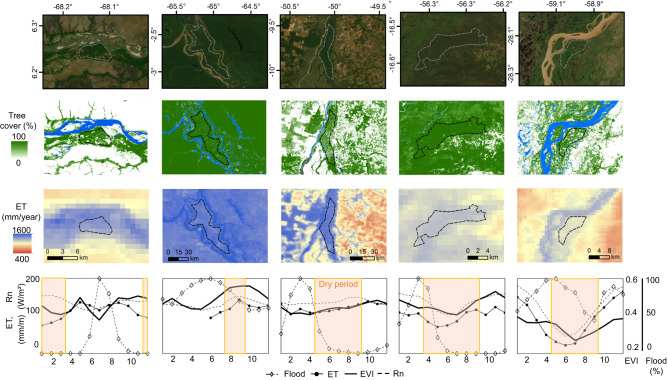
Evapotranspiration patterns and drivers in floodplain forests across South America. The figures present true color RGB composition (first row), tree cover fraction (second row), and annual evapotranspiration (ET) map (third row). In the fourth row, climatology of evapotranspiration, flood fraction and Enhanced Vegetation Index (EVI) variables are presented for the polygons in the map of the third row, and the dry period is defined according to precipitation regime. Dry period in the bottom figure is defined as months with precipitation smaller than 100 mm/month. Source of satellite images on second row: Source Esri, Digital Globe, GeoEye, Earthstar Geographics, CNES/Airbus DS, USDA, USGS, AeroGRID, IGN, and the GIS User Community.

While in the rest of the article we address the entire South American wetland areas, here we compare ET processes in five floodplain forests located from north to the south of the continent, using the MODIS Enhanced Vegetation Index (EVI; see Methods) as a proxy of stomatal activity and forest dormancy under flooding conditions^51–53^. Although the EVI signal may be affected by the inundation itself, its use here is reasonable given the high tree cover in the assessed floodplains. Both water excess and a water deficit can hinder wetland forest activity across South America, depending on plant adaptation and local-scale factors, including the soil’s water retention capacity. The highest ET rates occur during the wet season (or dry-wet transition in the Amazon “várzeas”, which corresponds to the floodplain leaf shedding period^54^ which occurs in many species) in all floodplains, while the flood peak leads to reduced vegetation activity or forest dormancy (low EVI) in all but the Paraná floodplain (Fig. 5). The phenological seasonality of the Paraná forests is predominantly driven by flooding, which occurs during the dry season (austral winter) due to the flood wave translation along the upstream river network, and its associated nutrient-rich sediments^18^. In the Paraná, the lowest EVI occurs during periods of receding waters, which correspond with periods of minimum energy availability, and EVI levels remain low until the onset of the wet season. In the Bananal forest, we observe that ET is not water-limited; in the adjacent floodable savannas, however, ET exhibits the opposite characteristic and decreases in the dry season (see the BAN flux tower in Fig. S9). A small—and, indeed, below the annual average—ET peak, which may be associated with soil evaporation,^33,51^ occurs in the month of maximum flooding in the Bananal and Pantanal floodplains. ET decreases in the Orinoco floodplains (i.e., densely vegetated floodplains as located in Fig. 5) during flooding; in contrast, the large-scale flooded savanna experiences its maximum ET during the flood period (Fig. 1), which suggests the greater importance of direct surface evaporation in this region associated with limited vegetation activity in riparian forests during flooding. Finally, the assessed Pantanal floodplain is a *Vochysia divergens* monodominant forest (the location where in situ ET data are available; see CAM tower in Fig. S9), which is not water-limited during the dry season due to plant adaptation strategies and exhibits a relatively high soil moisture content throughout the entire year^44,48^. In this case, the reduced vegetation activity observed during the dry season may be related more strongly to a reduction in available energy.

### Contrasting mechanisms in the Amazon floodplains

**Fig. 6:**
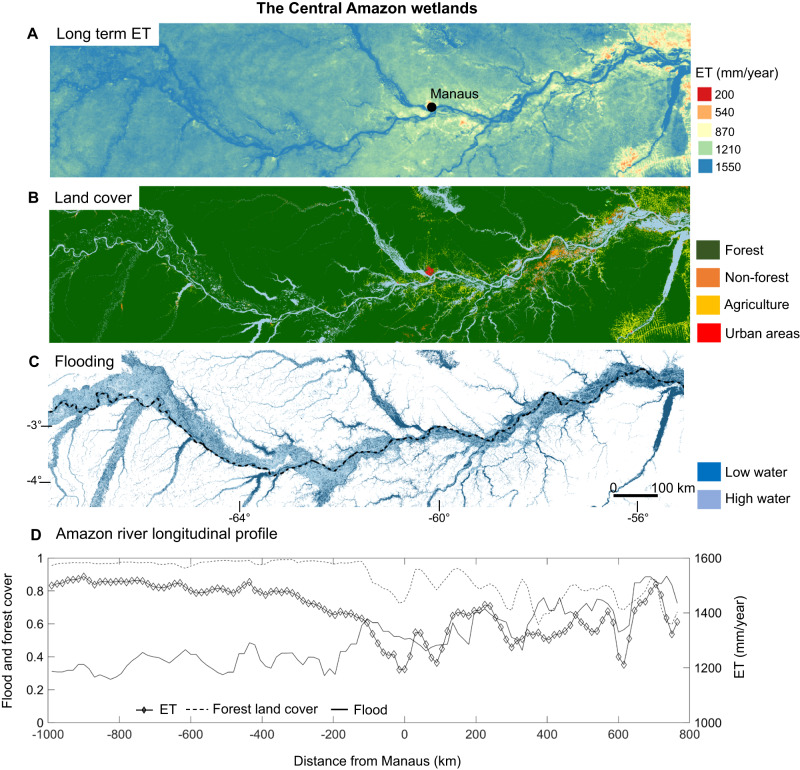
Spatial patterns of evapotranspiration across forest and non-forest areas in Amazon floodplain environments. **A** Long term evapotranspiration (ET) (2000-2015) across different land covers in the Central Amazon floodplains. The lower reaches are associated to larger cover of non-forest vegetation (savanna and grasslands) and higher flood fraction. **B** Land cover. Note that forest refers to both flooded and non-flooded areas. **C** Flooding (maximum and minimum inundation extent, considering high and low water periods derived from the LBA-ECO LC-07 Wetland Extent, Vegetation, and Inundation: Lowland Amazon Basin dataset - https://daac.ornl.gov/LBA/guides/LC07_Amazon_Wetlands.html; Hess et al.^94^). **D** River longitudinal profile showing ET, flood fraction and forest cover. Forest land cover data for the year 2009 from the Project MapBiomas - Collection 4.1 of Brazilian Land Cover & Use Map Series, accessed on 1^st^ Dec 2021 through the link https://plataforma.mapbiomas.org/.

In the Amazon River floodplain, the maximum wetland ET occurs at the transition between the dry and wet periods, which corresponds with maximum vapor pressure deficit, wind speed and E_0_ values. This pattern is similar to the ET pattern that occurs in the Amazon uplands^55^, where ET is energy-limited and strongly correlated with Rn, the highest values of which occur in the dry-wet period transition when cloud cover is limited. In addition, as discussed in the previous section, forest transpiration in flooded areas tends to be limited by flooding and ends up peaking during the dry season because of the river flood propagation process (i.e., there is a lag between precipitation and flooding peak). In contrast, the ET/E_0_ ratio peaks during maximum flooding in the floodplain areas in the Lower Amazon (Fig. S6), e.g., those downstream of Manaus. Overall, these areas have more flooding due to the existence of various floodplain lakes, thus highlighting the role of open water evaporation. On a large scale, two compensating effects interact to determine the actual annual ET in the Amazon floodplain (Fig. 6). First, more dense tree cover occurs in the upper reaches (roughly upstream from the city of Manaus; Fig. 6D), whereas the downstream reaches—with a large proportion of native herbaceous plants—are associated with lower precipitation rates, a longer dry season, smaller flood depths, less nutrient availability^56^, and the conversion of forest to agricultural areas and pastures^57^. Second, the downstream reaches have more floodplain lakes, with many permanently flooded areas, thus experiencing flooding for a longer period of the year, and both of these characteristics increase ET. The combination of these two opposing effects produces the highest annual ET rates in the upstream reaches, which exhibit greater tree cover, while a decreasing ET trend is observed in the downstream direction, with an exception (i.e., an increase) only in the furthest downstream parts that are subject to greater flooding (because of large permanent open water areas). The downstream Amazon surpasses the upstream region only during the high flood period (June and July) because of open water evaporation (Fig. S1).

## Discussion

Utilizing a comparative hydrology approach, our study provides insights on wetland ET processes on a continental scale. The interplay between climate, landscape geomorphology, soil, and vegetation drives ET patterns in South American wetlands. We confirm that wetlands largely affect the regional energy balance in temperate to tropical areas^39^, and thus have a major role on climate regulation. Wetlands act as a major source of water loss in South American basins by moving 13%–29% more water to the atmosphere than do the uplands adjacent to the Paraná, Iberá, Magdalena, Pantanal, and Pampas wetlands. While differences between the Amazon floodplain and adjacent uplands are not quite as distinct, an exception among equatorial wetlands occurs for the Magdalena Mompós depression in the transition between equatorial and tropical climates, which is associated with one of the most flooded environments in South America (i.e., a geomorphological factor) and is surrounded by non-forest vegetation with relatively low ET rates. Our results also highlight the role of flood propagation in the ET dynamics of river floodplains, especially for the Pantanal wetland, and they corroborate recent findings that flood dynamics drive the seasonality of the wetland vegetation activity^52^. While the strong seasonal variation of available energy (Rn) drives the overall magnitude of ET in temperate wetlands, seasonal ET variation in tropical and equatorial wetlands is more susceptible to variability in surface water availability and vegetation activity. In turn, the flood pulse generally enables wetland systems to meet the evaporative demand while depressing vegetation activity in all assessed floodplain forests, except for the Paraná River, where input of nutrient-rich sediments (originated in the Paraguay River basin) during flooding may increase vegetation activity. Although maximum ET occurs during the wet season in all floodplains, it does not coincide with maximum flooding, which generally hampers transpiration. These conclusions have implications for the development of earth system models, which can improve their predictive capabilities regarding surface-atmosphere interactions under future environmental changes by accurately representing wetland flooding dynamics and their impacts on wetland vegetation. For instance, the increased latent heat observed in our study has been shown to decrease precipitation rates in wetlands worldwide^22^. However, a proper understanding of such mechanisms, especially under the dense cloud cover of equatorial environments, requires further study. Although the implemented ET algorithm is limited in depicting local-scale patterns due to the misrepresentation of microtopography features, soil heterogeneity^58^, and dynamics of open water and macrophyte cover over flooded areas^27, 59^, the regional scale of our analysis and its validation with in situ data (Supplementary Note 2) support the suitability of the model adopted for this study. Similarly, the adopted inundation dataset does not resolve small-scale wetlands that may occur, although their impact on regional ET is arguably smaller than those of large wetland patches. Given the close relation between LST and surface water availability within the landscape, ET algorithms based on LST are considered more appropriate for estimating wetland ET than other remote sensing methods that depend upon vegetation indices and land cover maps^38,39^. Compared to other LST-based methods, the algorithm also exhibits low sensitivity to meteorological input data^60^. In addition, despite limitations regarding meteorological reanalysis data, our study focuses on a regional scale analysis, which errors related to local-scale patterns are minimized. On the other hand, estimates of Rn and VPD based on GLDAS reanalysis product were well aligned with others studies^21,33^. As they advance toward local-scale analyses, future studies should aim to fine-tune the ET calculation for each case by developing strategies of parameterization individually for each wetland.

Across South America, tropical and temperate wetlands face alternating cycles characterized by a range of soil conditions—from fully saturated to completely dry. Consequently, floodable savannas generally exhibit similar ET rates in relation to nearby forests during flooding but the similarity diminishes during the dry season. This finding has important implications for understanding the potential impacts of environmental changes since these ecosystems have evolved based on the feedback between the vegetation and the physical environment^61^. Changes in wetland flooding dynamics would largely affect the regional vegetation distribution and thus its energy partition. Furthermore, in hyperseasonal wetlands, associated with non-forest vegetation, groundwater appears to play a minor role, given the usual dry season water deficit^33^, while the opposite seems true in temperate grasslands such as the Pampas, as demonstrated by our analysis (Fig. 4B) and estimates by the NASA’s Gravity Recovery and Climate Experiment (GRACE) mission for the system’s slow water storage depletion^43^.

Because a two-way feedback system operates between wetland vegetation and physical flooding mechanisms, ET losses may serve as a regulatory feedback. This hypothesis has been suggested for the Pampas^43^ and the Pantanal wetlands in South America. In the latter, the system’s self-maintenance has been proposed to occur through tree expansion (especially *Vochysia divergens*) during extremely wet years^8^; these trees invade pastures^8^, exhibit higher growth and transpiration rates, and use water less efficiently than do non-dominant trees^44,48^. Our study demonstrates that these “super-dominant” tree species indeed alter the energy partition in the Northern Pantanal (forest in Fig. 5*)* towards higher water losses through ET; however, the long-term distribution of such species should be assessed in future research to better evaluate the ET regulation hypothesis. In highly dynamic reaches of the Amazon basin, after floodplain disturbances caused by sedimentation, some pioneer trees colonize the newly formed areas and can exhibit higher ET rates than non-pioneer trees in the first years of development^62^. In addition, the importance of regional-scale vegetation-atmosphere feedback in the Amazonian upland forests has been suggested because forest’s maintenance require high precipitation rates, while the forests’ ET is also responsible for downwind precipitation. In such cases, forest loss could further reduce precipitation and thereby accelerate the loss of additional forestlands^63^ while also reducing precipitation in downwind wetlands, such as the Pantanal^64^.

The vulnerability of wetlands to environmental changes must be better understood to ensure the sustainable provision of ecosystem services. For instance, agriculture expansion poses significant challenges for wetland conservation across South America^6^, and understanding the impacts of these changes on wetland ET remains challenging. While forest loss in upstream areas can increase water availability in wetlands located downstream^65^, the removal of trees may increase the availability of open water as well as encourage the colonization of new trees with higher ET rates. Fires pose a particular threat to floodplains, and this has recently been debated in the Amazon black-water floodplains (especially in the Negro River basin), which, during the dry season, may be more vulnerable to fires than are uplands due to the former’s lower leaf area index, more open canopy, and lower relative humidity^66–68^. In 2020, a large-scale drought affected the Pantanal, triggering the most impactful fires ever reported. These fires destroyed a large portion of the biome^10^, and their impact on vegetation will affect the wetland ET for years to come. Flood pulse alterations caused by dams in large river-floodplain systems (e.g., the Paraná, the Amazon, the Magdalena, and the Pantanal) also hold the potential to modify regional wetland ET dynamics and their associated vegetation, while interfluvial wetlands are less connected to rivers and thus less vulnerable. However, interfluvial areas, especially those close to deforested areas, are largely vulnerable to human activities such as wetland drainage. Finally, the potential effects of climate change on wetland ET and vegetation must be addressed. Although many floodplain trees have deep roots^21^, this does not apply to all floodplain tree species, and uncertainties remain regarding their ability to cope with the precipitation reductions (by some estimates, up to 20%^29,69^) and concomitant decreases in water availability that are projected to afflict equatorial and tropical South American wetlands by the end of the century^29,69,70^. Such decreases could lead equatorial wetlands to face a tropical climate regime, which would likely decrease ET. In turn, future research should assess the ability of savanna vegetation, which tends to have superficial roots, to cope with such challenges in hyperseasonal wetlands. Recent studies have suggested that severe droughts may affect floodplain tree photosynthesis and growth to a greater extent than do anomalous floods^62,71^. As research continues, the complex interplay between climate change—with a likely increase of vapor pressure deficit and CO_2_ concentrations^72^—and regional differences in available energy and water availability will ultimately define the fate of South American wetlands.

## Methods

### Remote sensing-based evapotranspiration

We implemented the Surface Energy Balance Algorithm for Land (SEBAL) model^41^ within Google Earth Engine (GEE) cloud computation environment, and a detailed explanation is provided in Supplementary Note 1 and in the study by Laipelt et al.^73^. Overall, SEBAL estimates instantaneous evapotranspiration as the residual of the surface energy balance (Eq. 1), using remote sensing and meteorological data (wind speed, specific humidity, surface air temperature, incoming shortwave radiation and atmospheric pressure) as input. The main model premise is that the near-surface vertical air temperature difference is linearly related to the surface temperature^41^, and that there are two extreme conditions that characterize the energy partitioning between sensible and latent heat. At the hot extreme condition, the latent heat is assumed as zero so that all available energy (${R}_{n}-G$) becomes sensible heat. Conversely, at the cold extreme condition all available energy becomes latent heat. To select the hot and cold endmember pixels, we used a simplified version adapted from the automated methodology from the METRIC model based on the Calibration using Inverse Modeling at Extreme Conditions (CIMEC) process^74^. The CIMEC process considers a population of candidate members based on quantiles of remote sensing estimations of Ts and NDVI to select the hot (dry) and cold (wet) pixels. The cold pixel is randomly selected among the coldest pixels (20th percentile of LST) of the most vegetated ones (95th percentile of NDVI), and the hot pixel considers the 80th percentile of LST and 10th percentile of NDVI^74^. To be consistent with the monthly inundation product (see ancillary data section below), we computed monthly evapotranspiration based on 8-day estimates.

1 $${LE}={R}_{n}-G-H$$

where ${LE}$ is the latent heat flux ($W.{m}^{-2}$), ${R}_{n}$ the net radiation ($W.{m}^{-2}$), $G$ the soil heat flux ($W.{m}^{-2}$), and $H$ the sensible heat flux (W.m^−2^).

Net radiation is computed as:

2 $${Rn}=\left(1-\alpha \right){{Rs}}_{{down}}+{{Rl}}_{{down}}-{{Rl}}_{{up}}-\left(1-{\varepsilon }_{0}\right){{Rl}}_{{down}}$$

where $\alpha$ is the broad-band surface albedo, ${{Rs}}_{{down}}$ the incoming short-wave radiation $(W.{m}^{-2})$, ${{Rl}}_{{down}}$ the incoming long-wave radiation $(W\,{m}^{-2})$, and ${{Rl}}_{{up}}$ the outgoing long-wave radiation $(W\,{m}^{-2})$.

${{Rs}}_{{down}},$ ${{Rl}}_{{down}}$ and ${{Rl}}_{{up}}$ were estimated following Allen et al.^75^.

Soil heat flux ($G$) is computed with the following equation, calibrated with remote sensing data and ground measurements at the flux towers.

3 $$G={R}_{n}({T}_{s}-273.15)\left(0.015\alpha \right)(1-0.8{\left({NDVI}\right)}^{1/3})$$

where ${T}_{s}$ is the land surface temperature ($K$), and $\alpha$ is the broad-band surface albedo. ${T}_{s}$ was obtained from the MODIS Land Surface Temperature and Emissivity dataset (MOD11).

The following equation is used to estimate the sensible heat flux ($H$):

4 $$H={\rho }_{{air}}{Cp}\frac{{dT}}{{r}_{{ah}}}$$

where ${\rho }_{{air}}$ is the air density (${kg}.{m}^{-3}$), ${Cp}$ the specific heat of air at constant pressure ($J.{{kg}}^{-1}{K}^{-1}$) and ${r}_{{ah}}$ the aerodynamic resistance (sm^−1^) between two near-surface heights, $z1$ and $z2$, where $z1$ = 0.1 and $z2$ = 2 m above the zero-plane displacement height. ${dT}$ is the near-surface temperature difference and represents a linear function of ${T}_{s}$, as proposed by Bastiaanssen et al.^41^:

5 $${dT}={{aT}}_{s}+b$$

where $a$ and $b$ are internally calibrated.

Since both $H$ and ${r}_{{ah}}$ are unknown, SEBAL adopts an iterative process. For the first iterative process, ${r}_{{ah}}$ is estimated assuming neutral stability:

6 $${r}_{{ah}}=\frac{{{{{\mathrm{ln}}}}}\left(z2/z1\right)}{{u}_{*}{k}\,}$$

where $z1$ and $z2$ are the heights above the zero-plane displacement of the vegetation where ${dT}$ are defined, ${u}_{*}$ the friction velocity ($m.{s}^{-1}$) and $k$ the von Karman’s constant (0.41).

The 8-day evapotranspiration (${{ET}}_{8-{day}}$) is computed with the following steps. Firstly, the 8-day evaporative fraction $(\Lambda )$ is calculated as:

7 $$\Lambda=\frac{LE}{{R}_{n}-G}$$

Then, ${{ET}}_{8-{day}}$ is calculated considering $\Lambda$ constant during the period of eight days. The 8-day net radiation (${{Rn}}_{8-{day}}$) was obtained by averaging the daily values.

8 $$E{T}_{8-day}=0.0864\,\Lambda \frac{R{n}_{8-day}}{\lambda }$$

The monthly evapotranspiration is finally computed as the average of all 8-day values within a given month.

### SEBAL input data and application for South American wetlands

SEBAL input data were based on the following products available in Google Earth Engine (GEE ID’s are provided):

- Surface Reflectance - MOD09A1.006 Terra Surface Reflectance 8-Day Global 500 m. GEE ID = MODIS/006/MOD09A1;
- Land Surface Temperature and Emissivity - MOD11A1.006 Terra Land Surface Temperature and Emissivity 8-Day Global 1 km. GEE ID = MODIS/006/MOD11A2;
- NDVI and EVI - MOD13A1.006 Terra Vegetation Indices 16-Day Global 500 m. GEE ID = MODIS/006/MOD13A1 (linearly interpolated to 8 days);
- Leaf Area Index (LAI) - MCD15A3H.006 MODIS Leaf Area Index/FPAR 4-Day Global 500 m. GEE ID = MODIS/006/MCD15A3H. For images between 2000-2002, a monthly average from 2003-2005 was used, given the unavailability of MODIS LAI data (linearly interpolated to 8 days);
- Meteorological input (wind speed, specific humidity, surface air temperature and incoming shortwave radiation): GLDAS 2.1^76^; GEE ID = NASA/GLDAS/V021/NOAH/G025/T3H;
- Digital Elevation Model (DEM) from SRTM9; GEE ID = USGS/SRTMGL1_003.

The criteria for the selection of endmember (hot and cold) pixels were based on a simplified method based on the CIMEC algorithm^74^. For each wetland, a MODIS image of 1 × 10^6 ^km² centered on the wetland was used to select the endmembers for calibration. MODIS data quality masks were used for each image, in addition to elevation masks (values lower than 600 m were assessed for all wetlands). For the Amazon River floodplains (Upstream and Downstream Amazon floodplains areas; see Fig. 1) and Negro interfluvial wetlands, the images from January to May were not considered given the persistent cloud cover in the region, which largely decreased data quality. Since we used MODIS 8-day (surface reflectance and LST) composites to estimate ET, we computed the 8-day average of the evaporative fraction ($\Lambda$) and Rn to estimate the 8-day ET. To compute monthly ET, we averaged the 8-day estimates existent in a given month to monthly time steps. The main SEBAL model output was monthly ET maps at 1 km spatial resolution for the period 2000–2015.

While the SEBAL methodology was already satisfactorily applied to individual wetlands worldwide, it was further validated here with 10 in situ monitoring sites located within or close to the assessed wetlands. The validation yielded a satisfactory accuracy of the ET seasonality across the continent, with a RMSE ranging between 0.4 and 1.2 mm.d^−1^ (Figs. S9 and S10), considering the 8-day average ET. Supplementary Note 2 presents more details on the model validation.

### SEBAL algorithm accuracy and limitations

The estimates of the SEBAL algorithm have been globally validated, and represent the state-of-the-art of the remote sensing methods for estimating ET. The obtained errors are in accordance with a known bias of 15–25% of flux towers measurements^77,78^. Despite the lack of measurements using flux towers in South America, multiple studies have demonstrated a satisfactory performance of the ET estimates from SEBAL^73,79–81^, including in wetlands conditions^60^. Regarding the suitability of the adopted ET algorithm for estimating ET in wetlands, LST-based models as SEBAL have been argued as the most adapted ones, given the effects of surface water on LST^38^. This is especially true if compared to models based on vegetation indices and land cover maps, which have a poor representation of surface water^77^. The applied SEBAL algorithm is the pioneer one and the most cited LST-based ET algorithm^82^, and the most used for wetlands so far, for both natural^60,83–88^ and artificial systems such as irrigated lands^88–90^. Overall, ET models based on the energy balance, including SEBAL, present known limitations, related for instance to the need of high quality images (with low presence of clouds) and meteorological information, as well as a poor representation of instantaneous soil heat dynamics, which typically corresponds to a small fraction of the energy balance in subtropical/tropical wetlands^1,7,28^. Moreover, underestimations may occur due to the non-representation of advection processes^41,91^, leading to an underestimation of the instantaneous latent heat by 10-20% under moist conditions^92^, which however results in a low impact in the evaporative fraction.

### Ancillary data

The developed ET dataset is conjointly analyzed with other state-of-the-art ancillary data at monthly time scale: GIEMS-2 monthly inundation at 25 km spatial resolution^42^, MSWEP precipitation^93^, GLDAS 2.1^76^ reanalysis data for Rn, vapor pressure deficit and wind speed, and MODIS LAI. Evaporative demand (E_0_) was computed with the FAO reference evapotranspiration equation, which was chosen for explicitly considering atmospheric variables as vapor pressure deficit and wind speed into the evaporative demand, allowing a consistent comparison among different locations.

### Experimental design

Each wetland polygon (Fig. 1) was defined as the GIEMS-2 maximum flood extent (i.e., the extent in terms of area) around the wetland location, considering a flood fraction (i.e., the long-term fraction of flooding in a given pixel) higher than 1%. The 25 km inundation fraction pixels were classified into two classes of floodability (50% most floodable, and 50% least flooded pixels), which defined the most floodable and least floodable areas depicted in the boxplots of Fig. S3. To avoid uncertain flood estimates in pixels with low flood fraction values, the analyses presented in Figs. 1–3 were performed for the set of the most flooded pixels. In addition, for each wetland the adjacent upland was identified as the non-flooded pixels within a 100 km buffer around the wetland polygon, randomly selected to have the same number of pixels as the most flooded pixels. The 2000–2015 period was adopted for being common to GIEMS-2, MSWEP and MODIS datasets. Linear correlations were performed among the estimated monthly ET, ET anomaly (normalized by monthly averages) and E_0_ and environmental variables to understand the drivers of ET processes (Fig. 3). The long term difference between wetland and adjacent upland ET was computed based on the long term (i.e., 2000–2015) average ET for each set of most flooded and upland pixels (Fig. 2).

### Supplementary information

Supplementary information

## Data Availability

A user-friendly Google Earth Engine application (available at https://etbrasil.users.earthengine.app/view/wetlands) was developed to facilitate the visualization of wetland variables (ET, LST, Rn, and LAI). MSWEP precipitation data are available at http://www.gloh2o.org. All SEBAL input data (MODIS images, GLDAS and DEM) are freely available through Google Earth Engine, and the developed SEBAL code is available at https://github.com/et-brasil/geeSEBAL.

## Code availability

Codes were written in MATLAB and Python and will be made available upon request by contacting the corresponding author.
