# Supplementary material for: Patterns and drivers of evapotranspiration in South American wetlands
Source: Nat Commun. 2023 Oct 20;14:6656. doi: 10.1038/s41467-023-42467-0 (PMC10589351; doi:10.1038/s41467-023-42467-0)
Supplement: Supplementary file 1 — Supplementary information [file 41467_2023_42467_MOESM1_ESM.pdf]

Site	Latitude	Longitude	Data availability	Land cover	Method	R^2	RMSE (mm.day^{-1})
**BDP**[1]	-16.497°	-56.411°	Jun/2011-Ago/2015	Woodland Savanna	Bowen ratio	0.76	1.00
**CAM**[1]	-16.555°	-56.286°	Jan/2007-Jan/2009	Riparian Forest	Bowen ratio	0.05	1.20
**FMI**[1]	-15.731°	-56.071°	Abr/2009-Mai/2013	Savanna mixed grassland-woodland	Bowen ratio	0.07	1.14
**K34**[2]	-2.609°	-60.209°	Jan/2000-Set/2006	Evergreen Broadleaf Forest	Eddy covariance	0.73	1.08
**K67**[3]	-2.856°	-54.958°	Jun/2002-Jan/2006	Evergreen Broadleaf Forest	Eddy covariance	0.02	0.40
**K83**[4]	-3.017°	-54.970°	Jun/2000-Mar/2004	Evergreen Broadleaf Forest	Eddy covariance	0.53	0.67
**NPW**[5]	-16.497°	-56.411°	Jan/2013-Sep-2016	Woodland Savanna	Eddy covariance	0.25	1.06
**TNR**	-12.831°	-69.283°	Jan/2017-May/2019	Mixed Forest	Eddy covariance	0.47	0.74
**VIR**[6]	-28.239°	-56.188°	Dec/2009-May/2012	Evergreen Needleleaf Forest	Eddy covariance	0.67	1.16
**BAN**[7]	-9.824°	-50.161°	Out/2003-Dez/2006	Woodland Savanna	Eddy covariance	0.04	0.85

Table S1 | Summary of the 10 micrometeorological sites used for model validation. Provided data are location, period of data availability, land cover type, method type, and performance metrics for the SEBAL model application (R^2 and RMSE). The main reference for each tower is cited together with the site name.

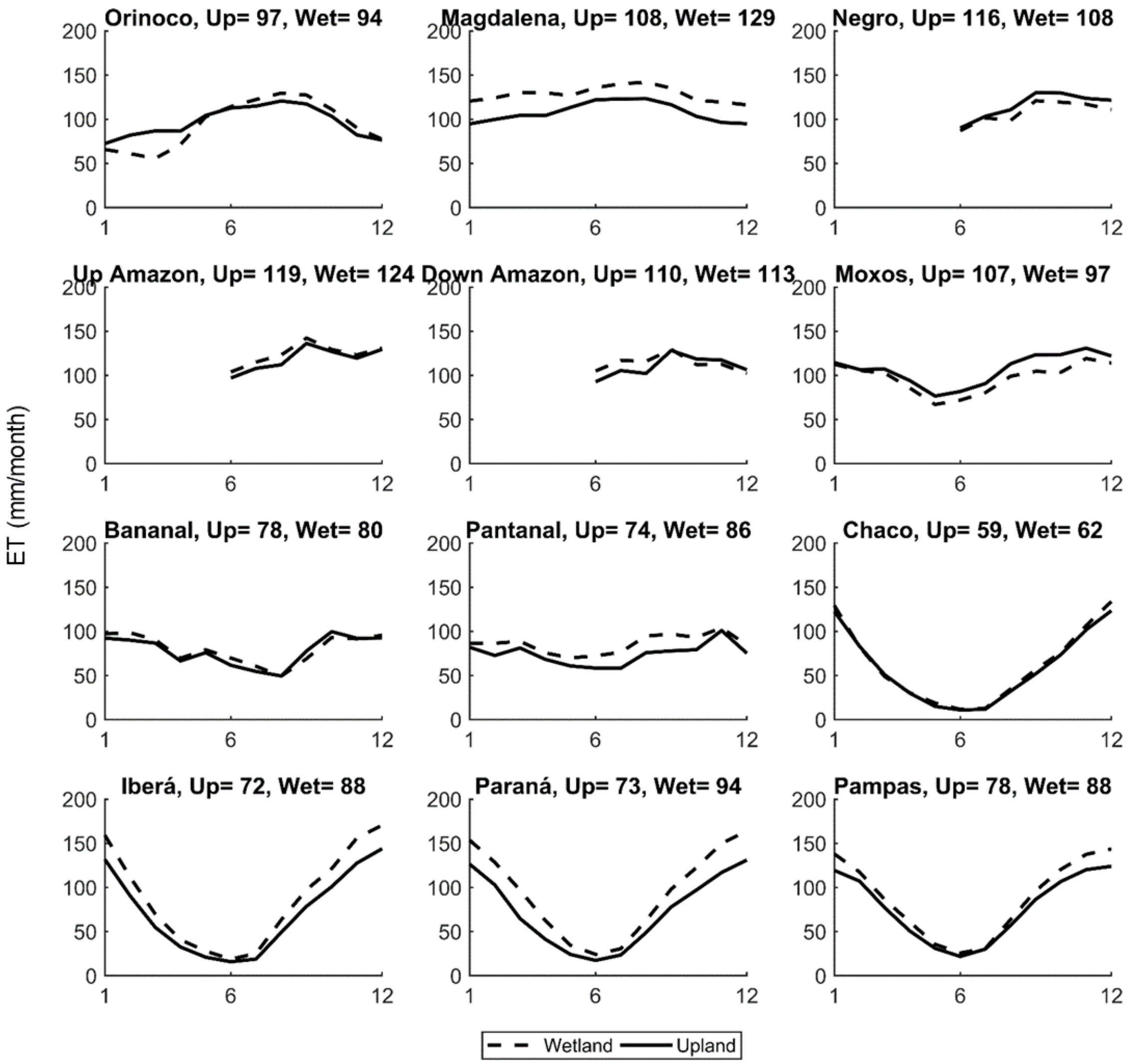

Figure S1 | Climatology of average evapotranspiration in the wetland and adjacent upland regions. The value in each figure's title relates to the long term average ET for upland (Up) and wetland (Wet). Please note that ET data for Negro, Up and Down Amazon are not computed for the months from January to May because of persistent cloud cover which hampers the use of MODIS optical data.

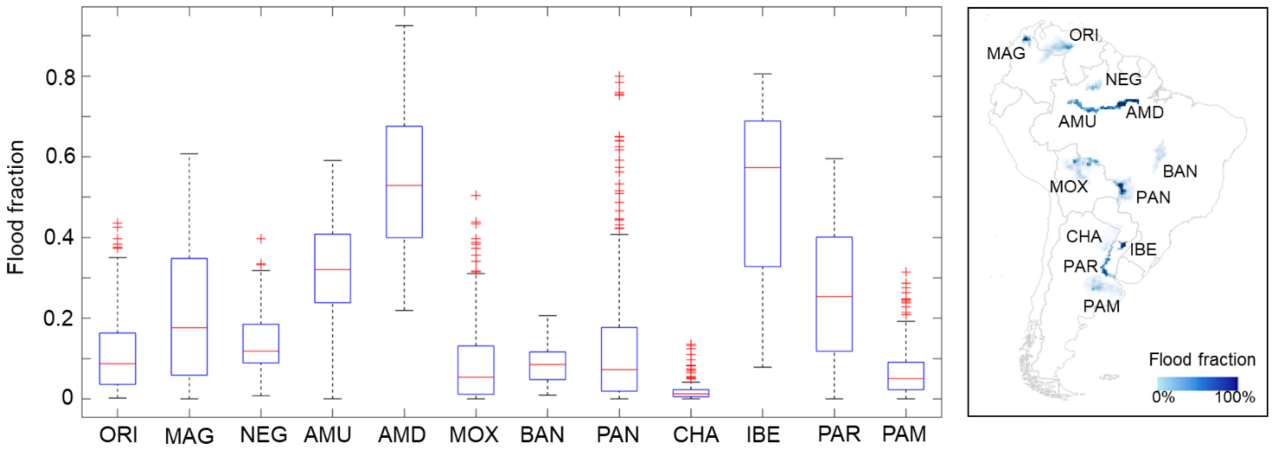

Figure S2 | Long term flood fraction for South American wetlands. Each boxplot refers to long term (2000-2015) flood fraction values for all 25 km GIEMS-2 pixels within each wetland area. Red crosses are outlier values.

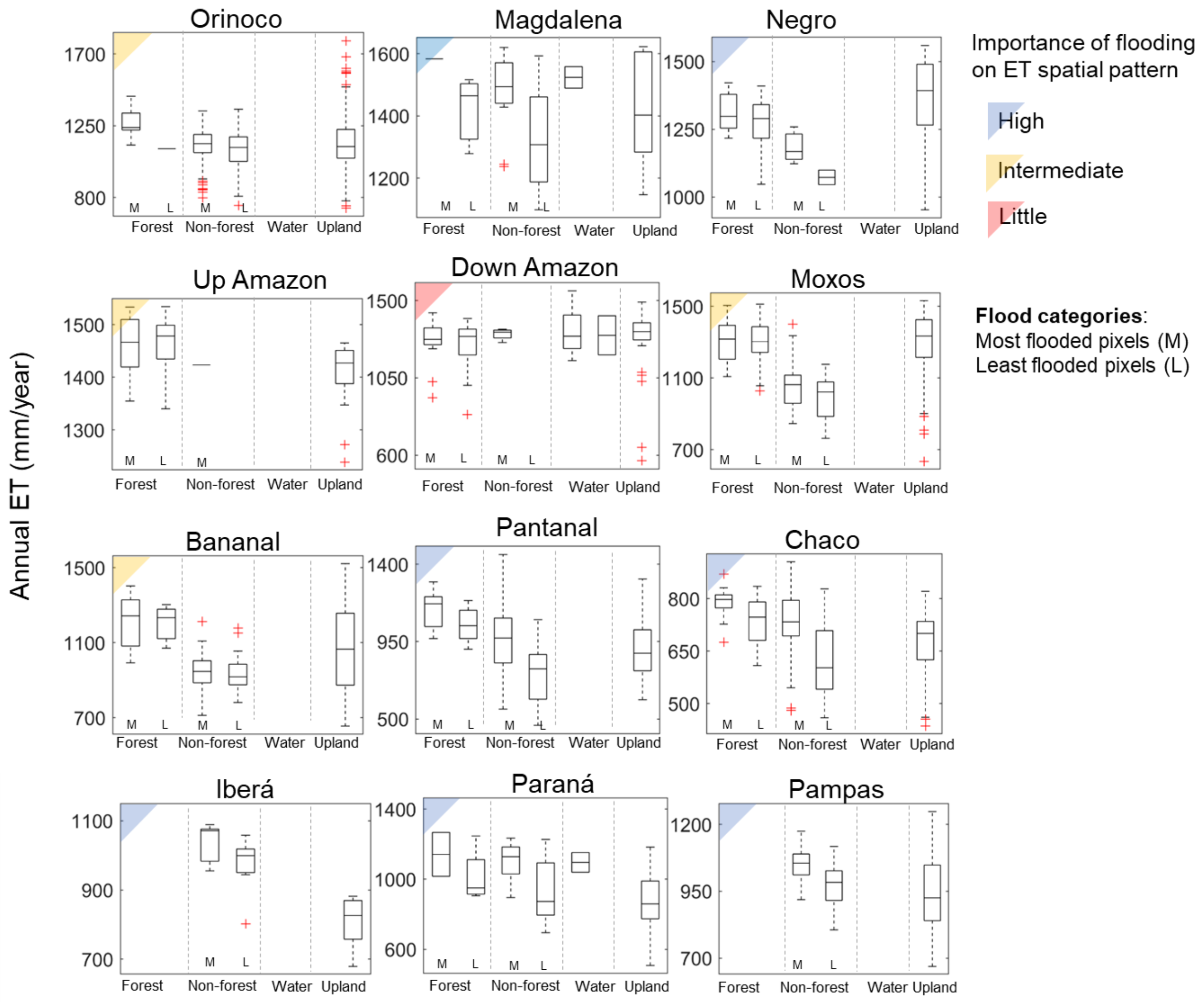

Figure S3 | Spatial assessment of annual evapotranspiration across South American wetlands. Each boxplot refers to all annual ET (long term average; 2000-2015) pixels within a given land cover (wetland forest, wetland non-forest, open water, upland) and a flood category (most flooded pixels (M) and least flooded pixels (L), based on 50%-50% quantiles) for each wetland area. High importance of flooding on ET spatial pattern: wetlands where the most flooded pixels have higher ET (median values) than the least flooded pixels. Intermediate: the most flooded non-forest pixels have higher ET than the least flooded ones, but lower ET than forest or upland; or the most flooded forest pixels have higher ET than uplands. Little: no major difference among wetland and upland ET.

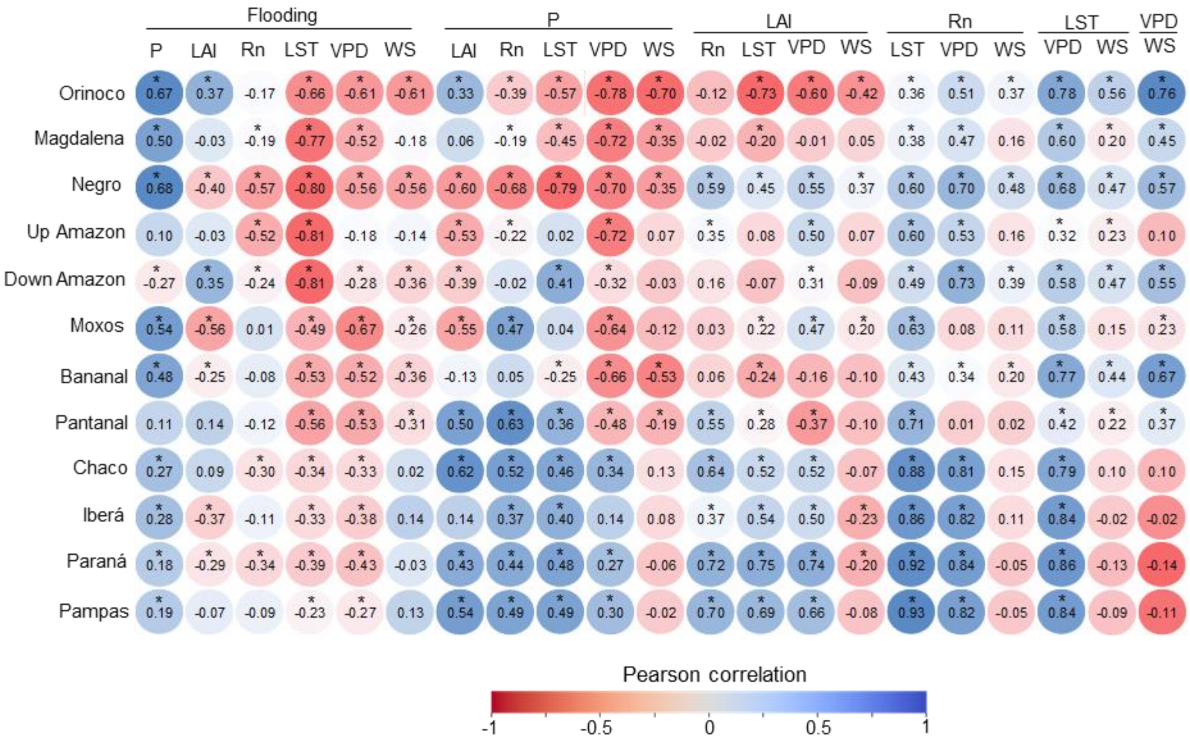

Figure S4 | Correlation among environmental drivers for the South American wetlands. Pearson correlation values are presented for the correlation among the following drivers for each wetland: flood fraction (FF), precipitation (P), leaf area index (LAI), net radiation (Rn), land surface temperature (LST), vapor pressure deficit (VPD) and wind speed (WS). *Significant correlations with P<0.01.

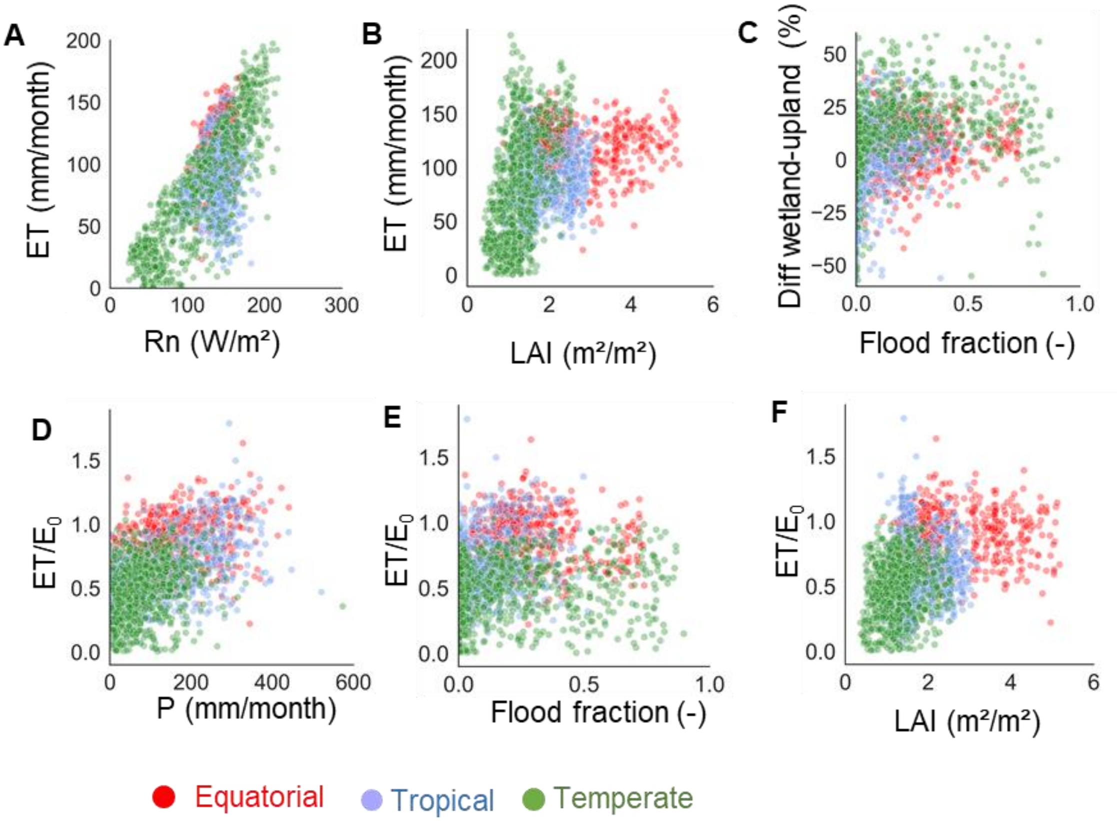

Figure S5 | Correlation between evapotranspiration (ET), ET to evaporative demand ratio (ET/E_0), and environmental variables for South American wetlands. The assessed environmental variables are net radiation (Rn), leaf area index (LAI), flood fraction and precipitation (P).

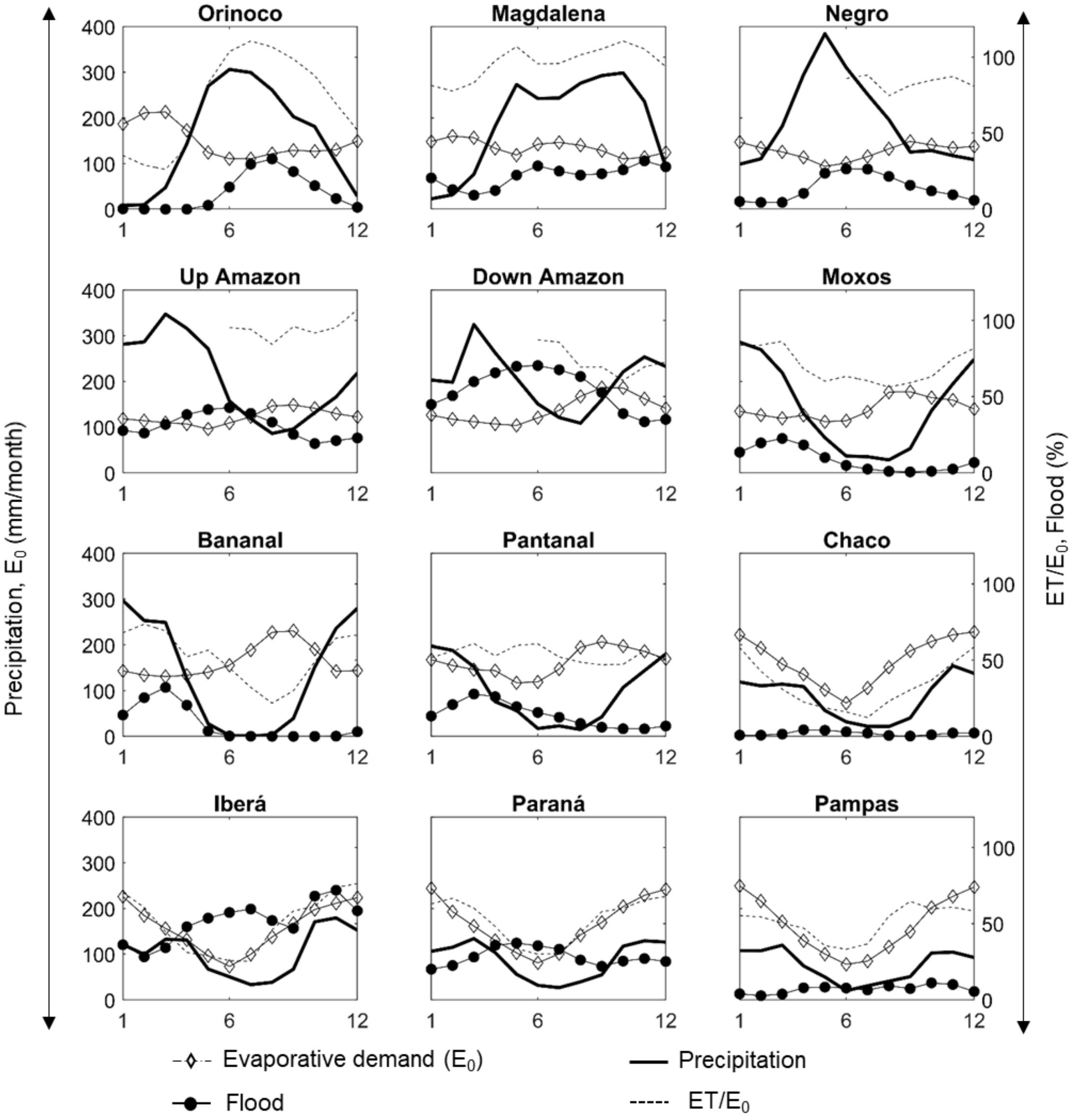

Figure S6 | Climatology of evaporative demand, flood fraction, precipitation, and evapotranspiration to evaporative demand (ET/E_0) ratio for the 12 wetlands. Please note that ET data for Negro, Up and Down Amazon are not computed for the months from January to May because of persistent cloud cover which hampers the use of MODIS optical data.

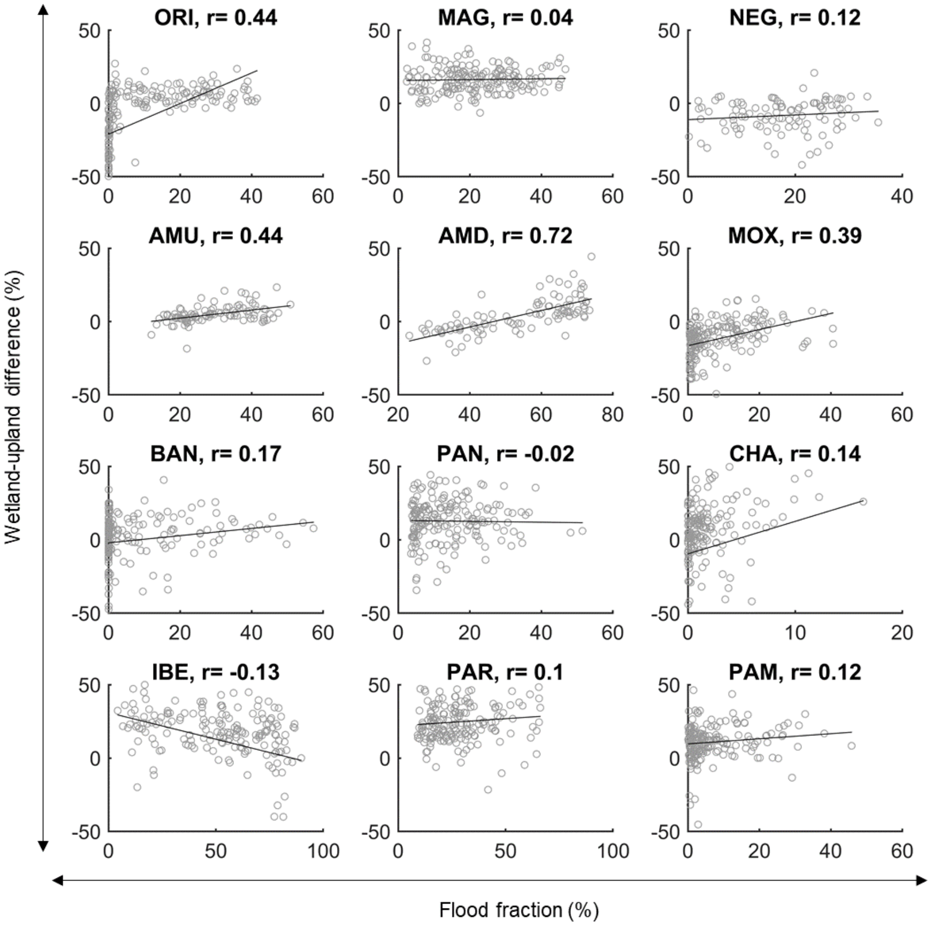

Figure S7 | Scatterplot between wetland-upland ET difference and flood fraction for each wetland. The Pearson correlation value is presented for each wetland.

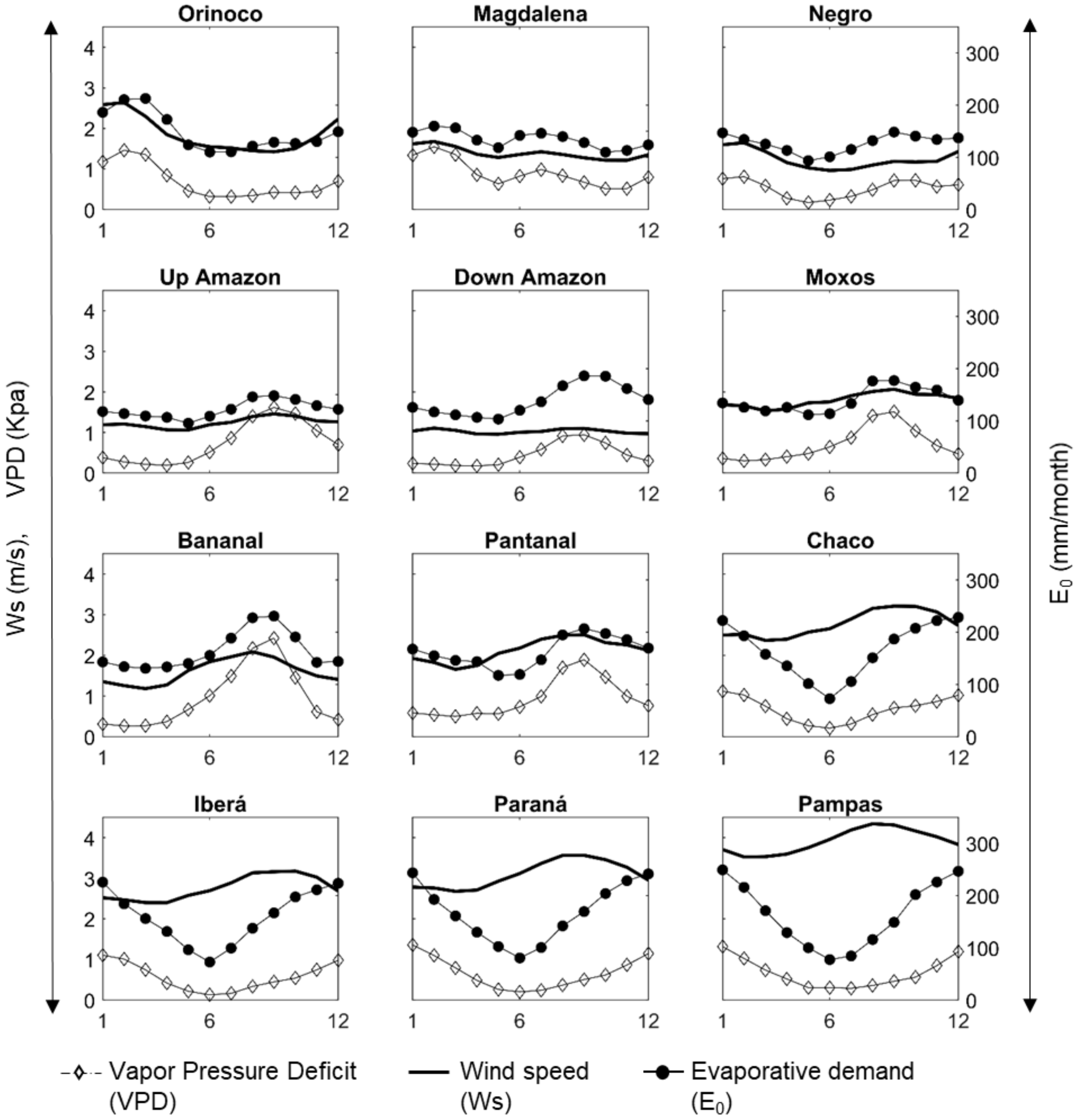

Figure S8 | Climatology of wind speed (Ws), vapor pressure deficit (VPD), and evaporative demand (E_0) for the 12 wetlands. E_0 generally follows the VPD pattern, and Ws to a lesser extent.

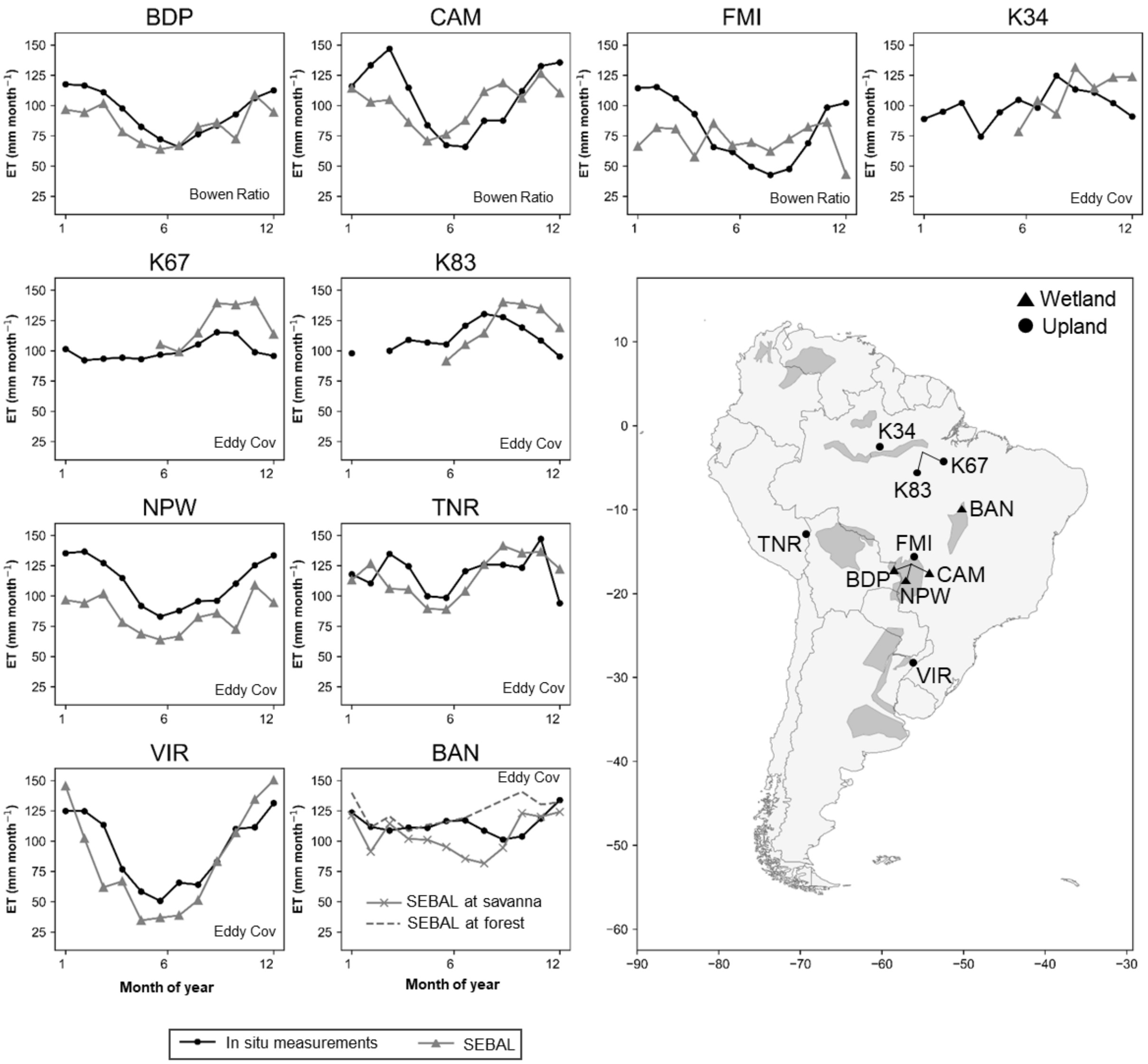

Figure S9 | Validation of the SEBAL-based evapotranspiration (ET) climatology with in situ data from 10 in situ micrometeorological towers (three using the Bowen ratio and seven the eddy covariance method) located across South America. Because of lack of available MODIS data for many months in the tropical/equatorial regions, the SEBAL climatology is based on the whole time series (2000-2015), while the in situ data have different periods of availability (Table S1). For the in situ measurements, monthly data were obtained by averaging all daily values in months with at least 75% of data available. In the map, towers located within wetlands are marked with a triangle, and those within uplands with a circle. For the BAN tower, SEBAL results are presented for two adjacent pixels (1 km far from each other), one located in a predominantly savanna and another in a mainly forest area, to show the differences in ET depicted by the model in this heterogeneous area. In this case, the tower likely receives contribution from both savanna and forest areas.

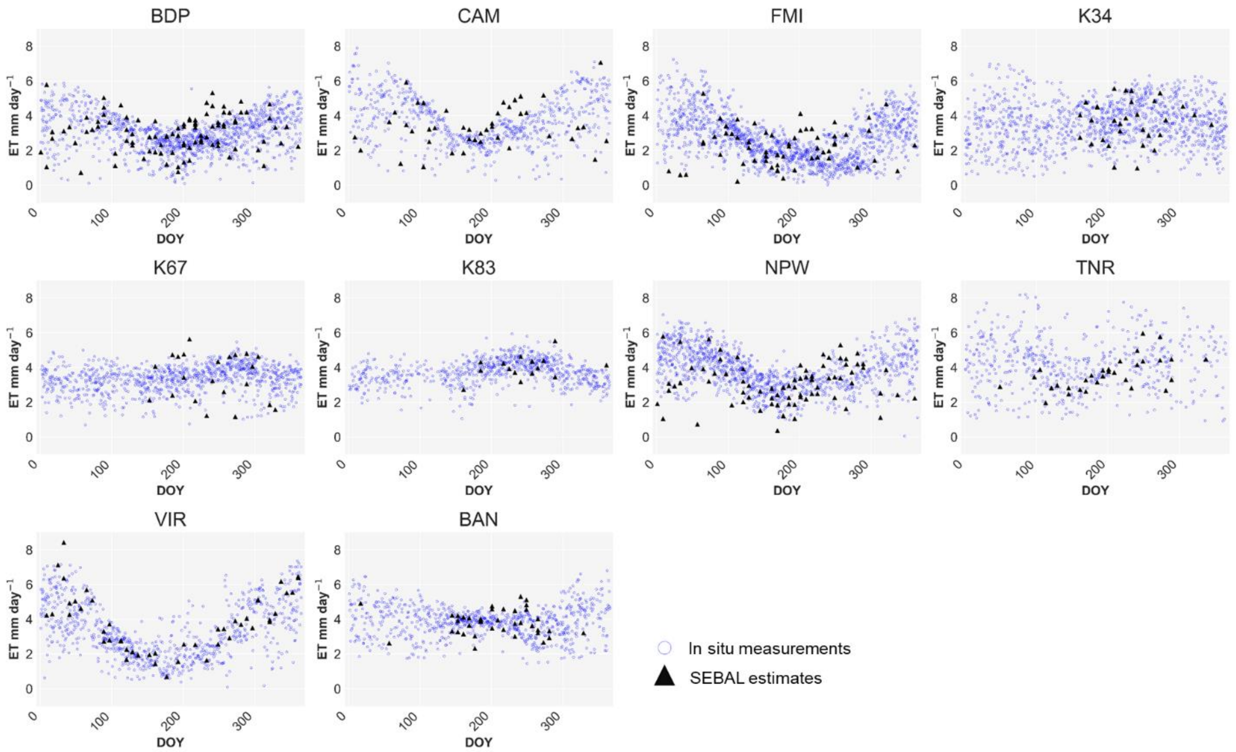

Figure S10 | Validation of SEBAL-based monthly evapotranspiration (ET) with in situ data. All daily in situ measurements for the 10 flux towers presented in Figure S9 are plotted for each day of the year, and compared to the SEBAL-based 8-day estimates for the same period of data availability.

Supplementary note 1. Detailed description of the SEBAL algorithm

The SEBAL algorithm[8] estimates the instantaneous evapotranspiration (or latent heat) rate as the residual of the surface energy balance (Equation 1), using remote sensing and meteorological data (wind speed, specific humidity, surface air temperature and incoming shortwave radiation).

$$LE = R_n - G - H \quad (1)$$

Where LE is the latent heat flux ($W\ m^{-2}$), R_n the net radiation ($W\ m^{-2}$), G the soil heat flux ($W\ m^{-2}$), and H the sensible heat flux ($W\ m^{-2}$).

The main model premise is that the near-surface vertical air temperature difference is linearly related to the surface temperature[9], and that there are two extreme pixels that characterize the landscape, namely the hot and cold pixels. At the hot pixel, the latent heat is assumed as zero so that all available energy ($R_n - G$) becomes sensible heat. Conversely, at the cold pixel all available energy becomes latent heat.

Our methodology adapts the SEBAL application by Laipelt et al[10] (originally using Landsat data) for MODIS imagery, and applied it within Google Earth Engine cloud computing environment (see SEBAL steps, as well as main input and output data in Figure S11). The instantaneous evapotranspiration rate estimated with Equation 1 is converted into 8-day evapotranspiration rate, which is the temporal resolution of the adopted MODIS products. This conversion is performed by assuming a constant evaporative fraction during the 8-day period. Finally, the 8-day values are averaged to yield monthly evapotranspiration.

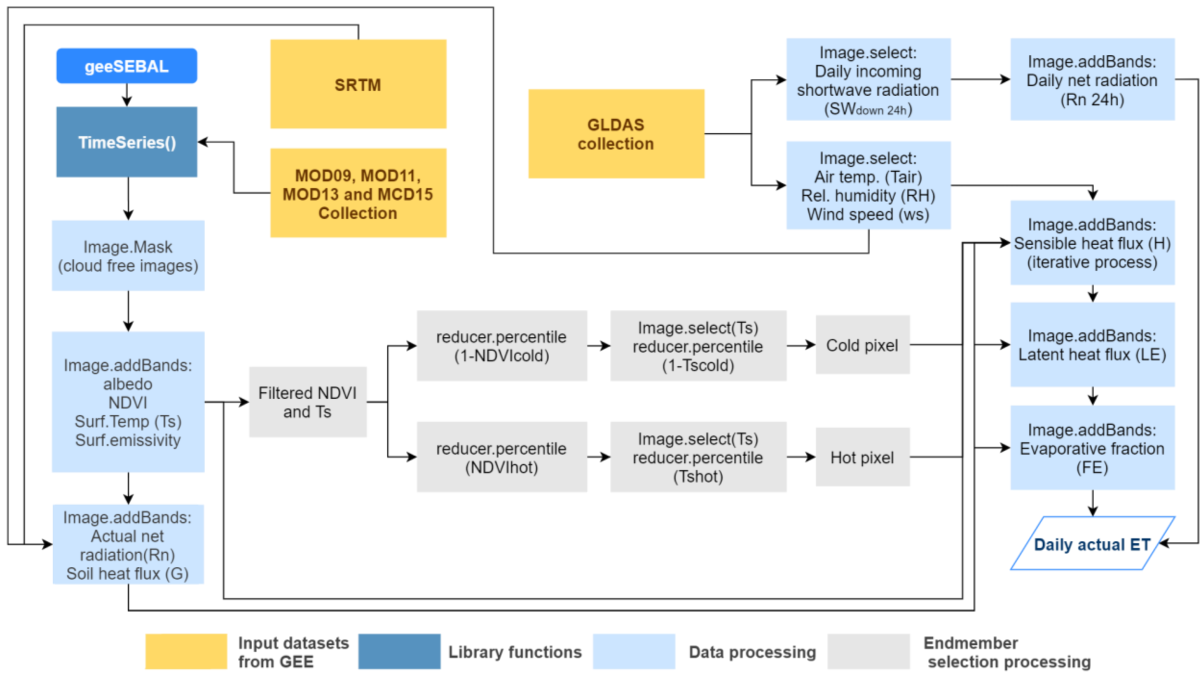

Figure S11 | Flow chart of SEBAL steps, and input and output data from the Google Earth Engine cloud computing environment.

Net radiation

Net radiation is calculated as:

$$Rn = (1-\alpha)Rs_{down} + Rl_{down} - Rl_{up} - (1-\varepsilon_0)\, Rl_{down} \quad (2)$$

where α is the broad-band surface albedo, Rs_{down} the incoming short-wave radiation ($W\ m^{-2}$), Rl_{down} the incoming long-wave radiation ($W\ m^{-2}$), and Rl_{up} the outgoing long-wave radiation ($W\ m^{-2}$).

Rs_{down}, Rl_{down} and Rl_{up} were estimated following Allen et al.[11]:

$$Rs_{down} = G_{sc} \cos(\theta_{rel})\, \tau_{sw}\, d^2 \quad (3)$$

$$Rl_{down} = 0.85(-\ln\, \tau_{sw})^{0.09}\, \sigma\, {T_s}^4 \quad (4)$$

$$Rl_{up} = \varepsilon_0\, \sigma\, {T_s}^4 \quad (5)$$

where G_{sc} is the solar constant ($1367\ W\ m^{-2}$), θ_{rel} the solar incidence angle, τ_{sw} the broad-band atmospheric transmissivity, d^2 the square of the eccentricity factor, σ the Stefan-Boltzmann constant ($5.67 * 10^{-9}\ W\ m^{-2} K^{-4}$), T_s the surface temperature (K) and ε_0 the broad-band surface emissivity.

$$\varepsilon_0 = 0.95 + (0.01 LAI) \quad (6)$$

The broad-band surface albedo (α) is calculated following Tasumi et al.[12]:

$$\alpha = \sum(\omega_\lambda * \rho_\lambda) \quad (7)$$

where ω_λ is a weighting coefficient and ρ_λ the surface reflectance.

To calculate τ_{sw}, we used the equation suggested by ASCE-EWRI[13]:

$$\tau_{sw} = 0.35 + 0.627 \exp\left[\frac{-0.00146P}{K_t \cos\theta_{hor}} - 0.075\left(\frac{W}{\cos\theta_{hor}}\right)^{0.4}\right] \quad (8)$$

where P is the atmosphere pressure (kPa), W the water in the atmosphere (mm), θ_{hor} the solar zenith angle over a horizontal surface, and K_t the unitless turbidity coefficient where $K_t = 1$ for clean air.

Atmospheric pressure is estimated as:

$$P = 101.3\left(\frac{293-0.0065z}{293}\right)^{5.26} \quad (9)$$

where z is the elevation above sea level (m) obtained from the SRTM mission.

Water in the atmosphere is estimated according to Garrison & Adler[14]:

$$W = 0.14\, e_a P + 2.1 \quad (10)$$

where e_a is the actual vapor pressure (kPa) estimated as[15]:

$$q = 0.622\frac{e_a}{P} \quad (11)$$

$\cos\theta_{hor}$ and d^2 equations are based on Duffie & Beckman[16]:

$$\cos\theta_{hor} = \sin(\delta)\sin(\varphi) + \cos(\delta)\cos(\varphi)\cos(\omega) \quad (12)$$

$$d^2 = 1 + 0.033\cos((DOY\ \ 2\pi)/365) \quad (13)$$

where $\theta_{hor}$is the solar zenith angle over a horizontal surface, and δ the declination of the Earth, δ = latitude of the pixel, ω = hour angle and DOY = day of year.

Soil heat flux

Soil heat flux (G) is computed with the following equation, calibrated with remote sensing data and ground measurements at the flux towers:

$$G = R_n(T_s - 273.15)(0.015\alpha)(1 - 0.8\,(NDVI)^{1/3}) \quad (14)$$

where T_s is the surface temperature (K).

Sensible heat flux

The following equation is used to estimate the sensible heat flux (H)[17]:

$$H = \rho_{air} Cp \frac{dT}{r_{ah}} \quad (15)$$

where ρ_{air} is the air density ($kg.m^{-3}$), Cp the specific heat of air at constant pressure ($J.kg^{-1}K^{-1}$) and r_{ah} the aerodynamic resistance (s m^{-1}) between two near-surface heights, $z1$ and $z2$, where $z1$ = 0.1 and $z2$ = 2 m above the zero-plane displacement height. dT is the temperature difference and represents a linear function of T_s, as proposed by Bastiaanssen et al.[9]:

$$dT = aT_s + b \tag{16}$$

where a and b are empirically determined coefficients.

Since both H and r_{ah} are unknown, SEBAL uses an iterative process. For the first iterative process, r_{ah} is estimated assuming neutral stability[11]:

$$r_{ah} = \frac{\ln(z2/z1)}{u_* \, k} \tag{17}$$

where z_1 and z_2 are the heights above the zero-plane displacement of the vegetation where dT are defined, u_* the friction velocity (m s^{-1}) and k the von Karman's constant (0.41).

To estimate u_* in the first iterative process, the following equation is used:

$$u_* = \frac{k \, u_{200}}{\ln(200/z_{om})} \tag{18}$$

where u_{200} is the wind speed (m s^{-1}) at 200m and z_{om} the momentum roughness length (m).

u_{200} is estimated as:

$$u_{200} = u_{*,ws} \, \frac{\ln\left(\frac{height}{z_{om}}\right)}{k} \tag{19}$$

where $u_{*,ws}$ is the friction velocity, and $height$ is assumed as 100 m[18].

$u_{*,ws}$ is estimated as:

$$u_{*,ws} = \frac{k \, u_x}{\ln\left(\frac{z_{ws}}{z_{om}}\right)} \tag{20}$$

where z_{ws} is the height of the GLDAS information and u_x the wind speed (m s^{-1}).

z_{om} is assumed as:

$$z_{om} = 0.12(h) \tag{21}$$

where h is the vegetation height (m), assumed as $h = 3$ m.

In the iterative process, dT_{hot} is the near-surface temperature difference at the hot pixel and is calculated with the following equation:

$$dT_{hot} = \frac{H_{hot} \, r_{ah\,hot}}{\rho_{hot} \, Cp} \tag{22}$$

where H_{hot}, $r_{ah\,hot}$ and ρ_{hot} are the sensible heat, aerodynamic resistance and air density at the hot pixel, respectively.

For H_{hot} and ρ_{hot} the following equations are used:

$$\rho_{hot} = -0.0046\, T_{s\ hot} + 2.5538 \quad (23)$$

$$H_{hot} = R_{n\ hot} - G_{hot} \quad (24)$$

where $T_{s\ hot}$, $R_{n\ hot}$ and G_{hot} are the land surface temperature, instantaneous net radiation and soil heat at the hot pixel, respectively.

To calculate the a and b coefficients of the linear relationship between T_s and dT, we consider dT_{cold} = 0 for the cold pixel (i.e., H_{hot}=0), which in combination with Equation 24 yields:

$$a = \frac{-dT_{hot}}{T_{s\ cold} - T_{s\ hot}} \quad (25)$$

$$b = dT_{hot} - a\, T_{s\ hot} \quad (26)$$

The next steps are the final part of the first iterative process. The Monin-Obukhov length (L) defines the stability conditions of the atmosphere in the iterative process. This equation represents the height at which forces of buoyancy and mechanical mixing are equal[11]:

$$L = -\frac{\rho_{air}\, Cp\, u*^3 T_s}{kgH} \quad (27)$$

where ρ_{air} is the air density (Kg m^{-3}), Cp the specific heat o fair at constant pressure (J Kg^{-1} K^{-1}), $u*$ the friction velocity (m s^{-1}), T_s the land surface temperature (K), k the von Karman's constant (0.41), g the gravitational acceleration (9.807 m s^{-2}) and H the sensible heat flux (W m^{-2}).

When L<0, the lower atmospheric boundary layer is unstable and when L>0, the boundary layer is stable. Momentum and heat transport ($\psi_m$and ψ_h) are computed using the following equations (following Paulson[19] and Webb[20]):

For L < 0:

$$\psi_{m\ (200\ m)} = 2\ln\left(\frac{1+x_{(200\ m)}}{2}\right) + \ln\left(\frac{1+x_{(200\ m)}^2}{2}\right) - 2\arctan\left(x_{(200\ m)}\right) + 0.5\pi \quad (28)$$

$$\psi_{h\ (2\ m)} = 2\ln\left(\frac{1+x_{(2\ m)}}{2}\right) \quad (29)$$

$$\psi_{h\ (0.1\ m)} = 2\ln\left(\frac{1+x_{(0.1\ m)}^2}{2}\right) \quad (30)$$

where:

$$x_{(200\ m)} = \left(1 - 16\frac{200}{L}\right)^{0.25} \quad (31)$$

$$x_{(2\ m)} = \left(1 - 16\frac{2}{L}\right)^{0.25} \quad (32)$$

$$x_{(0.1\ m)} = \left(1 - 16\frac{0.1}{L}\right)^{0.25} \quad (33)$$

For L>0:

$$\psi_{m\,(200\,m)} = -5\left(\frac{2}{L}\right) \quad (34)$$

$$\psi_{h\,(2\,m)=} -5\left(\frac{2}{L}\right) \quad (35)$$

$$\psi_{h\,(0.1\,m)=} -5\left(\frac{0.1}{L}\right) \quad (36)$$

In case of neutral conditions (L = 0), H=0, ψ_m=0 and ψ_h=0.

For the Equation 34, a value of 2 m is adopted instead of 200 m following the suggestion by Allen et al.[11], in order to avoid numerical instability.

Finally, r_{ah} and $u*$ are estimated again using the values obtained from $\psi_m$and ψ_h:

$$r_{ah} = -\frac{\ln(z_1/z_2)-\psi_{h(z_2)}+\psi_{h(z_1)}}{u*\,k} \quad (37)$$

$$u* = \frac{u_{200}\,k}{\ln(200/iz_{om})-\psi_{m\,(200\,m)}} \quad (38)$$

where iz_{om} is momentum roughness length for each pixel, which was based on the following equation, calibrated with remote sensing data and ground measurements at the flux towers:

$$iz_{om} = e^{((0.4\,(NDVI/\alpha)-2.4)} \quad (39)$$

The iterative process continues until the stability of stability conditions of the atmosphere is obtained and the absolute difference are lower than 0.1:

$$absolute\ difference = dT_{(n)} - dT_{(n-1)} + r_{ah\,(n)} - r_{ah\,(n-1)} \quad (40)$$

With the end of iterative process, we have a stable value of r_{ah}, and H is calculated using Equation 15.

Monthly evapotranspiration

The instantaneous latent heat is computed using the energy balance equation:

$$LE = R_n - G - H \quad (41)$$

The 8-day evapotranspiration (ET_{8-day}) is then computed with the following steps. Firstly, the evaporative fraction (Λ) is calculated:

$$\Lambda = \frac{LE}{R_n-G} \quad (42)$$

Then, the latent heat of vaporization (λ) (kJ kg^{-1}) is estimated as:

$$\lambda = 2.501 - 0.002361\,(T_s - 273.15) \quad (43)$$

The instantaneous evapotranspiration rate is computed as:

$$ET_{inst} = 0.0036\frac{LE}{\lambda} \tag{44}$$

ET_{8-day} is then calculated considering Λ constant during the 8-day period, while the 8-day net radiation ($R_{n,8-day}$) is estimated by averaging the daily net radiation ($R_{n\,24h}$) over eight days, which in turn is computed following de Bruin[21]:

$$R_{n_{24h}} = (1-\alpha)Rs_{down\ 24h} - C_S(\frac{Rs_{down\ 24h}}{Ra_{24h}}) \tag{45}$$

where $Rs_{down\ 24h}$ is the daily mean incoming shortwave radiation obtained from GLDAS 2.1 ($W\ m^{-2}$), C_S a constant set to 110, and Ra_{24h} the extraterrestrial radiation for a 24-hour period ($W\ m^{-2}$).

$$ET_{8-day} = 0.0864\ \Lambda\frac{R_{n,8-day}}{\lambda} \tag{46}$$

The monthly evapotranspiration is finally computed as the average of all 8-day values within a given month.

Supplementary note 2. Suitability of the SEBAL algorithm for evapotranspiration computation over wetlands

The estimates from SEBAL algorithm are validated against 10 in situ flux towers for many years (Table S1; Figs. S9 and S10), which provide a massive amount of measured data considering data scarcity in South America. It includes four towers within the wetland areas and six in uplands close to the analyzed wetlands. Estimates from seven flux towers are based on the eddy covariance method, and three on the Bowen ratio method. Table S1 presents detailed information on the 10 towers.

Overall, the algorithm has a satisfactory performance for most flux towers, with Root Mean Square Error (RMSE) varying from 0.4 to 1.2 mm.day^{-1}, which is within the error range for most of the ET models used worldwide. The coefficient of determination (R^2) is greater than 0.45 for five towers, and ET seasonality is generally well captured (Fig. S10). In the case of the Bananal wetland (BAN), the flux tower is located within a savanna-forest mosaic and this local landscape's heterogeneity makes the ET estimate to be sensitive to the analyzed pixel (1 km resolution). Thus, if looking at two adjacent pixels the ET may become water-limited during the dry season (for savanna) or not (for forest). For the BAN tower, both ET pixels are shown in Fig. S9. Additionally, differences in the climatology, as seen in Fig. S9, may arise from the different period of analysis between the in situ (available period provided in Table S1) and SEBAL data (climatology for 2000-2015).

Multiple studies have demonstrated a satisfactory performance of the ET estimates from SEBAL[22–25], and LST-based models as SEBAL tend to better represent the effects of surface water if compared to models based on vegetation indices and land cover maps[26]. In addition, SEBAL also has a low sensitivity to input meteorological data, in comparison to other LST-based methods available at global scale (e.g., SSEBOp and METRIC) that are dependent on reference evapotranspiration estimates. This advantage makes SEBAL more suitable for poorly gauged areas as the South American wetlands. Given the overall satisfactory performance of SEBAL when compared to in situ data, and the suitability of its conceptualization, we conclude that SEBAL is today one of the most appropriate algorithms to be employed for the characterization of wetland ET dynamics.

To further demonstrate the suitability of SEBAL in comparison to other ET algorithms, we performed additional analyses using GLEAM and MOD16 products, which are among the most widely used ET datasets (Figures S12 and S13). These two algorithms are not based on LST but mainly rely on vegetation indices to estimate land surface properties and feed ET algorithms. They are driven by meteorological reanalysis as an indicator of water availability (e.g., vapor pressure deficit and available radiation for MOD16, and precipitation and available radiation for GLEAM), relying on vegetation phenology as surface processes[27]. In addition, vegetation-based models are also dependent on biome properties look-up tables and land cover classifications, therefore most of the uncertainties reported are related to the low spatial resolution of meteorological reanalysis and land cover parameterization[28], with higher uncertainties in areas with wetlands and high soil moisture[29,30] and higher accuracy over large and homogeneous areas[28]. Consequently, they are known to have difficulties to estimate differences between wetland and adjacent uplands, which can be depicted by LST-based algorithms such as SEBAL and other relatively similar algorithms like SSEBOP[10,23,31]. Using both MOD16 and GLEAM, we performed the same Budyko-like framework done in our analysis with SEBAL. Figure S13 clearly shows the lack of representation of the regional effect of inundated lands on ET for MOD16 and GLEAM as compared to SEBAL. Similarly, the comparison of the three different ET estimates with the measurements over the 10 flux towers (see Figure S13) shows that SEBAL also has relatively better performance metrics (R^2 and RMSE) than the other two datasets. These analyses support the choice of LST-like algorithms, as SEBAL, for estimating wetland ET at large domains.

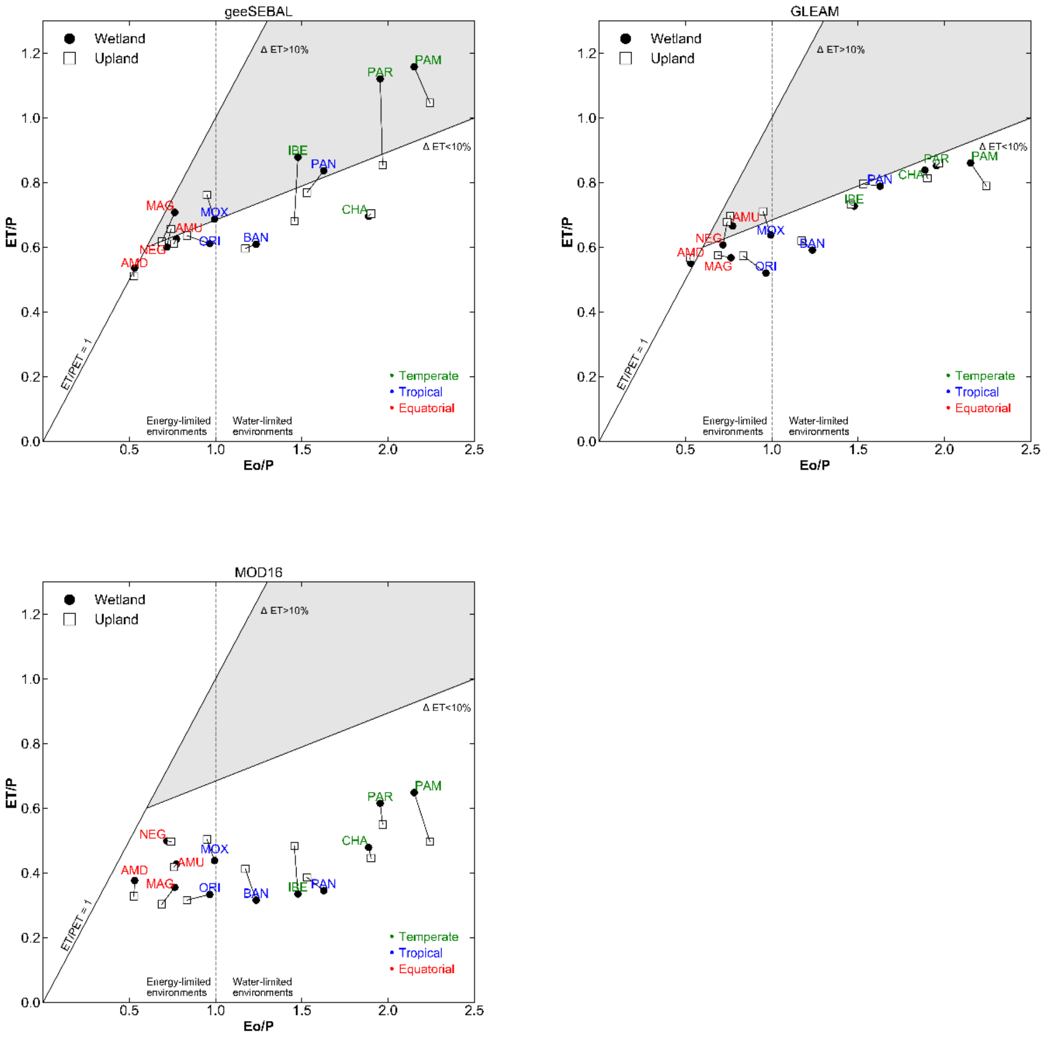

Figure S12 | Budyko-live assessment of various wetlands across South America, for three different remote sensing based ET datasets.

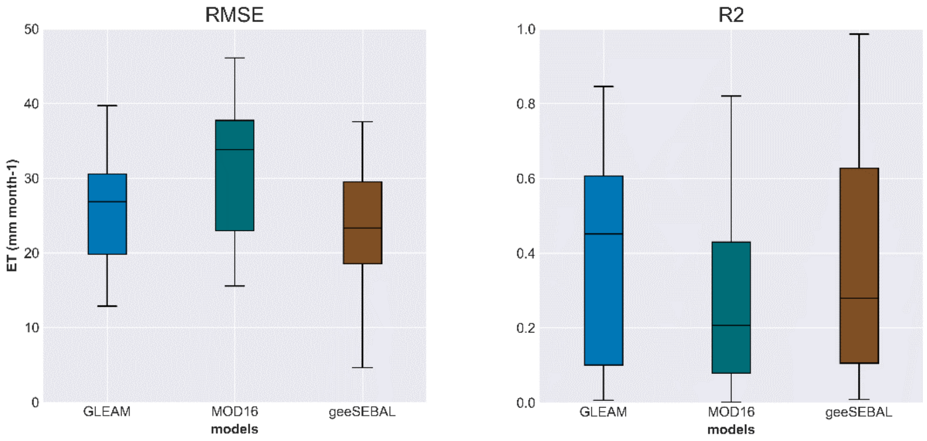

Figure S13 | Performance of three ET datasets for 10 in situ flux towers across South America.

Supplementary References

1. Biudes, M. S. *et al.* Patterns of energy exchange for tropical ecosystems across a climate gradient in Mato Grosso, Brazil. *Agric. For. Meteorol.* **202**, 112–124 (2015).
2. Araújo, A. C. *et al.* Comparative measurements of carbon dioxide fluxes from two nearby towers in a central Amazonian rainforest: The Manaus LBA site. *J. Geophys. Res. Atmos.* **107**, LBA 58-1-LBA 58-20 (2002).
3. Saleska, S. R. *et al.* Carbon in Amazon Forests: Unexpected Seasonal Fluxes and Disturbance-Induced Losses. *Science (80-.).* **302**, 1554–1557 (2003).
4. Goulden, M. L. *et al.* Diel and seasonal patterns of tropical forest CO2 exchange. *Ecol. Appl.* **14**, 42–54 (2004).
5. Dalmagro, H. J. *et al.* Radiative forcing of methane fluxes offsets net carbon dioxide uptake for a tropical flooded forest. *Glob. Chang. Biol.* **25**, 1967–1981 (2019).
6. Posse, G., Lewczuk, N., Richter, K. & Cristiano, P. Carbon and water vapor balance in a subtropical pine plantation. *iForest - Biogeosciences For.* **9**, 736–742 (2016).
7. Borma, L. S. *et al.* Atmosphere and hydrological controls of the evapotranspiration over a floodplain forest in the Bananal Island region, Amazonia. *J. Geophys. Res. Biogeosciences* **114**, 1–12 (2009).
8. Bastiaanssen, W. G. M., Menenti, M., Feddes, R. A. & Holtslag, A. A. M. A remote sensing surface energy balance algorithm for land (SEBAL). 1. Formulation. *J. Hydrol.* **212**–**213**, 198–212 (1998).
9. Bastiaanssen, W. G. M. G. M. *et al.* A remote sensing surface energy balance algorithm for land (SEBAL): 1. Formulation. *J. Hydrol.* (1998) doi:10.1016/S0022-1694(98)00253-4.
10. Laipelt, L. *et al.* Assessment of an Automated Calibration of the SEBAL Algorithm to Estimate Dry-Season Surface-Energy Partitioning in a Forest–Savanna Transition in Brazil. *Remote Sens.* **12**, 1108 (2020).
11. Allen, R., Tasumi, M. & Trezza, R. Satellite-Based Energy Balance for Mapping Evapotranspiration With Internalized Calibration (METRIC) – Model. *J. Irrig. Drain. Eng.* **133**, (2007).
12. Tasumi, M., Allen, R. G. & Trezza, R. At-Surface Reflectance and Albedo from Satellite for Operational Calculation of Land Surface Energy Balance. *J. Hydrol. Eng.* **13**, 51–63 (2008).
13. ASCE. *The ASCE Standardized Reference Evapotranspiration Equation*. *The ASCE Standardized Reference Evapotranspiration Equation* (2005). doi:10.1061/9780784408056.
14. Garrison, J. D. & Adler, G. P. Estimation of precipitable water over the United States for application to the division of solar radiation into its direct and diffuse components. *Sol. Energy* **44**, 225–241 (1990).
15. Shuttleworth, W. J. *Terrestrial Hydrometeorology*. (2012). doi:10.1002/9781119951933.
16. Duffie, J. A. & Beckman, W. A. *Solar Engineering of Thermal Processes: Fourth Edition*. *Solar Engineering of Thermal Processes: Fourth Edition* (2013). doi:10.1002/9781118671603.
17. Allen, R. *et al.* Satellite-based ET estimation in agriculture using SEBAL and METRIC. *Hydrol. Process.* **25**, 4011–4027 (2011).
18. Waters, R., Allen, R., Tasumi, M., Trezza, R. & Bastiaanssen, W. *SEBAL, Surface Energy Balance Algorithms for Land. Advance Training and Users Manual. Idaho: a NASA EOSDIS/Synergy grant from the Raytheon Company University of Idaho.* http://www.posmet.ufv.br/wp-content/uploads/2016/09/MET-479-Waters-et-al-SEBAL.pdf (2002).
19. Paulson, C. A. The Mathematical Representation of Wind Speed and Temperature Profiles in the Unstable Atmospheric Surface Layer. *J. Appl. Meteorol.* **9**, 857–861 (1970).
20. Webb, E. K. Profile relationships: The log-linear range, and extension to strong stability. *Q. J. R. Meteorol. Soc.* **96**, 67–90 (1970).
21. Bruin, H. A. . De. *From penman to makkink*. *Evaporation and Weather: Technical Meeting 44, Ede, …* vol. 39 (1987).
22. Laipelt, L. *et al.* Long-term monitoring of evapotranspiration using the SEBAL algorithm and Google Earth Engine cloud computing. *ISPRS J. Photogramm. Remote Sens.* **178**, 81–96 (2021).

23. Kayser, R. H. *et al.* Assessing geeSEBAL automated calibration and meteorological reanalysis uncertainties to estimate evapotranspiration in subtropical humid climates. *Agric. For. Meteorol.* **314**, (2022).
24. Santos, C. A. G., Silva, R. M. da, Silva, A. M. & Brasil Neto, R. M. Estimation of evapotranspiration for different land covers in a Brazilian semi-arid region: A case study of the Brígida River basin, Brazil. *J. South Am. Earth Sci.* **74**, 54–66 (2017).
25. Moletto-Lobos, I., Mattar, C. & Barichivich, J. Performance of Satellite-Based Evapotranspiration Models in Temperate Pastures of Southern Chile. *Water* vol. 12 (2020).
26. Biggs, T. *et al.* Remote Sensing of Actual Evapotranspiration from Cropland: Chapter 3. in *Remote sensing handbook, Vol. III: Remote sensing of water resources, disasters, and urban studies* (ed. Thenkabail, P. S.) (CRC Press, 2015).
27. Biggs, T. W., Marshall, M. & Messina, A. Mapping daily and seasonal evapotranspiration from irrigated crops using global climate grids and satellite imagery: Automation and methods comparison. *Water Resour. Res.* **52**, 7311–7326 (2016).
28. Li, X. *et al.* Evapotranspiration Estimation for Tibetan Plateau Headwaters Using Conjoint Terrestrial and Atmospheric Water Balances and Multisource Remote Sensing. *Water Resour. Res.* **55**, 8608–8630 (2019).
29. Souza, V. de A. *et al.* Evaluation of MOD16 Algorithm over Irrigated Rice Paddy Using Flux Tower Measurements in Southern Brazil. *Water* **11**, 1911 (2019).
30. Yang, Y. *et al.* Comparison of three dual-source remote sensing evapotranspiration models during the MUSOEXE-12 campaign: Revisit of model physics. *Water Resour. Res.* **51**, 3145–3165 (2015).
31. Long, D., Singh, V. P. & Li, Z.-L. How sensitive is SEBAL to changes in input variables, domain size and satellite sensor? *J. Geophys. Res. Atmos.* **116**, (2011).
